# Cellular communication network factor 1 promotes retinal leakage in diabetic retinopathy via inducing neutrophil stasis and neutrophil extracellular traps extrusion

**DOI:** 10.1186/s12964-024-01653-3

**Published:** 2024-05-16

**Authors:** Ting Li, Yixia Qian, Haicheng Li, Tongtong Wang, Qi Jiang, Yuchan Wang, Yanhua Zhu, Shasha Li, Xuemin He, Guojun Shi, Wenru Su, Yan Lu, Yanming Chen

**Affiliations:** 1 Department of Endocrinology and Metabolism, Guangdong Provincial Key Laboratory of Diabetology, Guangzhou Key Laboratory of Mechanistic and Translational Obesity Research, The Third Affiliated Hospital of Sun Yat-sen University, Guangzhou, 510630 China; 2 Department of Clinical Immunology, The Third Affiliated Hospital of Sun Yat-sen University, Guangzhou, 510630 China; 3 Department of Ocular Immunology & Uveitis, State Key Laboratory of Ophthalmology, Zhongshan Ophthalmic Center, Guangdong Provincial Key Laboratory of Ophthalmology and Visual Science, Sun Yat-sen University, Guangzhou, 510515 China

**Keywords:** Diabetic retinopathy, Retinal inflammation, CCN1, Retinal leakage, Blood-retinal barrier, Neutrophils, Neutrophil extracellular traps

## Abstract

**Background:**

Diabetic retinopathy (DR) is a major cause of blindness and is characterized by dysfunction of the retinal microvasculature. Neutrophil stasis, resulting in retinal inflammation and the occlusion of retinal microvessels, is a key mechanism driving DR. These plugging neutrophils subsequently release neutrophil extracellular traps (NETs), which further disrupts the retinal vasculature. Nevertheless, the primary catalyst for NETs extrusion in the retinal microenvironment under diabetic conditions remains unidentified. In recent studies, cellular communication network factor 1 (CCN1) has emerged as a central molecule modulating inflammation in pathological settings. Additionally, our previous research has shed light on the pathogenic role of CCN1 in maintaining endothelial integrity. However, the precise role of CCN1 in microvascular occlusion and its potential interaction with neutrophils in diabetic retinopathy have not yet been investigated.

**Methods:**

We first examined the circulating level of CCN1 and NETs in our study cohort and analyzed related clinical parameters. To further evaluate the effects of CCN1 in vivo, we used recombinant CCN1 protein and CCN1 overexpression for gain-of-function, and CCN1 knockdown for loss-of-function by intravitreal injection in diabetic mice. The underlying mechanisms were further validated on human and mouse primary neutrophils and dHL60 cells.

**Results:**

We detected increases in CCN1 and neutrophil elastase in the plasma of DR patients and the retinas of diabetic mice. CCN1 gain-of-function in the retina resulted in neutrophil stasis, NETs extrusion, capillary degeneration, and retinal leakage. Pre-treatment with DNase I to reduce NETs effectively eliminated CCN1-induced retinal leakage. Notably, both CCN1 knockdown and DNase I treatment rescued the retinal leakage in the context of diabetes. In vitro, CCN1 promoted adherence, migration, and NETs extrusion of neutrophils.

**Conclusion:**

In this study, we uncover that CCN1 contributed to retinal inflammation, vessel occlusion and leakage by recruiting neutrophils and triggering NETs extrusion under diabetic conditions. Notably, manipulating CCN1 was able to hold therapeutic promise for the treatment of diabetic retinopathy.

**Supplementary Information:**

The online version contains supplementary material available at 10.1186/s12964-024-01653-3.

## Introduction

Diabetic retinopathy (DR) stands as one of the most prevalent complications of diabetes mellitus (DM) and poses a significant threat to vision [1]. In 2020, DR was reported as the fifth most prevalent cause of blindness among individuals aged 50 years and older [2]. Pathologically, the increased infiltration of neutrophils and heightened adhesion between neutrophils and the endothelium result in retinal inflammation and vessel leukostasis, contributing to the progression of DR [3]. To date, studies have confirmed a positive correlation between diabetic retinopathy and both the neutrophil-to-lymphocyte ratio (NLR) and the neutrophil percentage-to-albumin ratio (NAR) in peripheral blood [4]. NETosis is a specific cell death process of neutrophils [5] and is characterized by the release of neutrophil extracellular traps (NETs), which are extracellular structures composed of chromatin and proteins such as myeloperoxidase (MPO), citrullinated histone H3 (Cit-H3), and neutrophil elastase (NE) [6]. NETs hallmarks including NE and DNA-histone complexes were identified as independent risk factors for DR [7]. Beyond peripheral blood, NETs have been detected in both vitreous bodies [8] and retina [9] of DR patients. The components of NETs cause vascular dysfunction by promoting retinal cells’ oxidative stress, senescence [10], apoptosis [11], thrombosis [12], inflammation pathway activation [13, 14], and wrecked cell-to-cell integrity [15]. As a result, the increased trafficking of leukocytes in capillaries and the presence of NETs ultimately lead to the disruption of the blood-retinal barrier (BRB) [13]. These findings underscore the importance of identifying the key molecule that precisely modulates NETs extrusion for the treatment of DR at different disease stages.

Cellular communication network factor 1 (CCN1), also known as cysteine-rich protein 61, is a matricellular protein secreted by various cell types including endothelial cells [16]. CCN1 is recognized for its involvement in a multitude of cellular processes, encompassing proliferation, differentiation, angiogenesis, apoptosis, and the formation of the extracellular matrix [17]. A previous study has unveiled elevated CCN1 levels in the vitreous humor of DR patients when compared to individuals without diabetes-related ocular conditions [18–20]. The upregulation of CCN1 in the retinas was also reported in diabetic mice [20–22] and rats [18]. Notably, intravitreal injection of anti-CCN1 antibody has been confirmed to reduce retinal neovascularization [23]. Beyond its role in promoting angiogenesis, CCN1 is now emerging as a pivotal molecular player in the modulation of inflammation under pathological conditions [24–27]. A growing body of research considers CCN1 as a regulator in immune cell trafficking, which attracts and locally immobilizes immune cells including monocytes [28–30], macrophages [24, 31], leukocytes [27] and lymphocytes [32]. Moreover, CCN1 has been reported to stimulate the production of reactive oxygen species (ROS) through the activation of Rac1 and NADPH oxidase (NOX) 2 [27]. In line with this, our prior research has demonstrated that CCN1 stimulates ROS production by activating NOX4 in DR [21]. Considering the well-established critical role of NOX/ROS activation in NETs extrusion [33–35], the aforementioned evidence suggests a potential connection between CCN1 and neutrophils. Yet, the mechanism underlying the interaction between CCN1 and neutrophils remains largely unclear and necessitates further investigation.

In this study, we delved into the intricate interplay between CCN1 and neutrophils and examined their roles in exacerbating retinal leakage in the context of diabetes. Our study cohort unveiled a marked elevation of circulating CCN1 in DR patients. Notably, circulating CCN1 was positively correlated with several key clinical parameters, including the absolute neutrophil count, NLR, NE, and the duration of diabetes. Mechanistically, CCN1 promoted neutrophil stasis within microvasculature by enhancing the adhesion, migration, and NETs extrusion of neutrophils. Significantly, CCN1 knockdown effectively counteracted the retinal leakage of diabetic mice. In summary, our study uncovers the substantial role played by CCN1 in retinal inflammation and retinal leakage by promoting neutrophil stasis and NETs extrusion during the progression of DR.

## Materials and methods

### Study participants and blood sample collection

This study was approved by the Third Affiliated Hospital of Sun Yat-sen University Network Ethics Committee following the principles of the Helsinki Declaration. Individuals with diabetes or healthy adults were respectively recruited from the Department of Endocrinology and Metabolism and Physical Examination Center of the Third Affiliated Hospital of Sun Yat-sen University, Guangzhou, China, in 2023. In addition, healthy adults were enrolled with written informed consent.

The study population comprised 76 consecutive patients with diabetes and 12 volunteers without chronic disease history between 18 and 70 years old. Of 76 patients with diabetes, 27 patients with diabetic retinopathy, and 49 patients without retinopathy were enrolled in the study. The diagnosis of diabetes is based on the 1999 World Health Organization (WHO) criteria [36]. The 7-field color fundus photographs were taken by a trained ophthalmic technician using VISUCAM Lite Digital Fundus Camera (Carl Zeiss Meditec AG, Jena, Germany) to diagnose diabetic retinopathy. Patients were excluded from this study if they had a history of renal failure with estimated glomerular filtration rate (eGFR) < 30 mL/min; acute infectious disease at the time of evaluation; a history of malignancy, mental disorders, autoimmune diseases, or severe heart or liver dysfunction; and history of solid or hematological neoplasia or active neoplasia. Those who had a history of eye diseases were also excluded. For each patient, a personal interview was conducted to collect basic demographic data regarding age, sex, height, and body weight. Information about medical history, duration of diabetes (years), diabetes treatment, and chemobiological examination results were also collected. Peripheral blood was collected in sodium citrate tubes (Becton Dickinson, San Jose, CA, USA). Whole blood samples were centrifuged for 15 min at 1550 g to isolate plasma for further analysis.

### Human peripheral blood and mouse neutrophil isolation

Isolation of neutrophils from human whole blood and mouse bone marrow was performed with MojoSort™ Whole Blood Human Neutrophil Isolation Kit (Biolegend#480152, California, United States) and MojoSort™ Mouse Neutrophils Isolation Kit (Biolegend#480057, California, United States) according to the manufacturer’s instructions.

Briefly, the steps are as follows. Incubation: The sample containing neutrophils (human blood or mouse bone marrow) is mixed with magnetic particles coated with an antibody cocktail comprising a mixture of multiple specific antibodies that recognize blood cells other than neutrophils. Binding: blood cells recognized by antibody cocktail selectively bind to the surface of the magnetic particles via antibody-antigen interactions. Magnetic Separation: The sample is placed in a magnetic field, causing the magnetically labeled blood cells to migrate towards the tube wall, while neutrophils remain in suspension. Collection: The supernatant containing purified neutrophils is collected, leaving those blood cells bound to the tube wall. Purity verification: Take a portion of the supernatant for flow cytometry experiments, using CD16 (Biolegend#360715, 1:200) and CD66b (Biolegend#305109, 1:200) to identify human neutrophils and CD11b (Biolegend#101225, 1:200) to identify mouse neutrophils. Samples with neutrophil purity greater than 90% will be used for downstream experiments.

### Mice and diabetes modeling

Mice Conventional Specific Pathogen Free (SPF) NOD/ShiLtJ mice and wild-type C57BL/6J mice were purchased from Guangdong Province Medical Experimental Animal Center. All mice were bred and maintained at the animal facility of Sun Yat-sen University under specific pathogen-free conditions. Mice were group housed in a controlled environment under the 12-h cycles of light-darkness, with free access to water and a standard chow diet. Mice were randomly assigned to each experimental group. The animal experiment was approved by the Animal Care Committee assigned by Sun Yat-sen University.

For NOD/ShiLtJ mice, 25-week-old female mice with random blood glucose levels above 16.7 mmol/L were assigned to the experiment group, and the age-matched female mice (with random blood glucose levels below 16.7 mmol/L) were assigned to the control group. As for C57BL/6J mice, 6-week-old male mice were used in animal experiments. For the modeling of diabetes, multiple low doses of STZ (55 mg/kg, Sigma-Aldrich, Steinheim, Germany), dissolved in citrate buffer (0.1 M, pH = 4.7), were intraperitoneally injected into animals (STZ-group) for continues 5 days. Meanwhile, the control group received an equal 0.1 M citrate buffer volume. Random blood glucose levels above 16.7 mmol/L were considered as diabetes one week after a 5-day injection.

### Intravitreal injection

The intravitreal injection was operated as reported [37]. In our study, recombinant CCN1 protein (MedchemExpress, NJ, USA) was expressed in HEK 293 cells system. In intravitreal injection, 2 µL volume of rCCN1 (10 µg/mL) or vehicle control (PBS) was applied. Injection of rCCN1 was operated on day 1 and day 4, and the retina was dissected on day 7 and day 14 respectively.

Lentivirus overexpressed CCN1 (LV-CCN1, pSLenti-CMV-CCN1-3xFLAG-PGK-Puro-WPRE) was synthesized and purified by OBIO Technology (Shanghai, China) Corp.Ltd. and the titer was 4.57E + 08/mL. In intravitreal injection, 2 µL of virus was applied to overexpress CCN1 in the mouse eye. The overexpression efficiency was confirmed on day 14. Further experiments were performed on day 30 and day 60. To knock down CCN1 in the mouse eyes, lentivirus-silenced CCN1 (LV-siCCN1, target sequence: CTTCTACAGGCTGTTCAAT) and vehicle (LV-Con, target sequence: TTCTCCGAACGTGTCACGT) were synthesized by GENECHEM Technology (Shanghai, China) Corp.Ltd. The titer was 2E + 8/mL and the volume used in intravitreal injection was 2 µL per eye. Intravitreal injection was performed at 12 weeks after diabetes modeling. The left eye of the mice accepted LV-Con injection, and the right eye accepted LV-siCCN1 injection. Mice were sacrificed one month after the virus interfered.

For the DNase I treatment experiment, 2 µL (1 U/µL) of DNase I (Thermo#EN0521, Massachusetts, USA) or vehicle control (PBS) was intravenously administered to mice 30 min before rCCN1 treatment on day 1 and day 7. Mice were sacrificed on day 14 and the retina was collected. In STZ-DM mice, 2 µL of DNase I was administered to the left eye, and an equal volume was administered to the right eye 8 weeks after diabetes modeling. The intravitreal injection was performed once per week for continuously 4 weeks, and the mice were sacrificed 1 week after the 4th injection.

### Evans blue permeability assay

Evans Blue (EB, Sigma-Aldrich#E2129, Missouri, USA) solution, with a concentration of 4.5 mg/mL, was administered to mice via tail vein injection at a dosage of 45 mg/kg. The mice were then placed on thermal blankets for 2 h following the injection. For visualization of EB leakage, eyes were then enucleated, fixed for 1 h in 4% PFA, and dissected to collect the retina for flat-mount. To quantify EB leakage, the mice were perfused with 20 mL of PBS to clear EB in vessels after 2-h circulation on thermal blankets. Retinas were dissected and weighed. Then retinal tissues were homogenized and sonicated in 150 µL of formamide per retina, followed by a 65 °C-metal bath for 18 h. The lysates were centrifuged, and the supernatant was used for EB quantification with a BioTek Synergy H1(excitation at 620 nm, emission at 680 nm).

### Immunofluorescence staining

For the retina paraffin sections, samples were dewaxed and antigen-retrieved before staining. For the retina frozen sections, samples were restored to room temperature before staining. For retina flat-mount, retinas were treated as reported [38]. Briefly, eyeballs were enucleated from mice and then fixed in 4% PFA made up in 2X PBS at room temperature for 20 min. Retinas were dissected, flattened, and fixed again by cold methanol for 20 min at -20 °C. Samples were blocked and permeabilized in 10% goat serum and 0.1% triton-X for 1 h at room temperature and then incubated with primary antibodies at 4 °C overnight. Secondary antibodies conjugated fluorescence were used to incubate samples for 1 h at room temperature. Finally, samples were applied with mounting medium with DAPI (Abcam, MA, USA). Between each step, samples were washed with PBS 3 times. Slides were photographed under confocal microscopy (Leica TCS SP5, Wetzlar, Germany). Quantification was determined from 3 to 4 non-overlapping fields per sample and the average was used for statistics in every experiment. Both fluorescence intensity and related areas were analyzed with ImageJ software.

The antibodies used for immunofluorescence staining were listed as follows: CCN1 (Abcam#24448, 1:200), Ly6G (Biolegend#108401, 1:400), IL-1b (Cell Signaling Technology#12242S, 1:500), MPO (Thermo Fisher Scientific#MA1-34067, 1:400), MPO (Cell Signaling Technology#79623, 1:400), NE (Cell Signaling Technology#89241; 1:200), Collagen IV (Sigma Aldrich#SAB4500375, 1:500), CD31 (Cell Signaling Technology#3528S, 1:800), GFAP (Cell Signaling Technology#3670, 1:1000), Histone H3 (citrulline R2 + R8 + R17) (Abcam#5103, 1:400), PAD4 (Thermo Fisher Scientific# PA5-18318,1:200), Iba1 (Abcam#5067, 1:800), goat anti-mouse conjugated with Alexa Fluor®555 (Cell Signaling Technology#4409, 1:1000), goat anti-mouse conjugated with Alexa Fluor®647 (Abcam#150115, 1:1000), goat anti-rabbit conjugated with Alexa Fluor®555 (Cell Signaling Technology#4413, 1:1000), goat anti-mouse conjugated with Alexa Fluor®488 (Abcam#150077, 1:1000). Other staining reagent: Isolectin GS-IB4 (Invitrogen#I21411, 1:200).

### ELISA

The concentrations of CCN1 (#EK10933) and NE (#EK1447) in the plasma of human peripheral blood were assayed with ELISA kits (Signalway Antibody, Maryland, USA) according to the manufacturer’s instructions.

### Western blot

Western Blot was performed according to our previous study [39]. Briefly, retinal tissues were homogenized and sonicated and lysates were analyzed by SDS-PAGE (10% acrylamide). Specific primary antibodies were used to incubate the membranes overnight at 4 °C. Secondary antibodies were used to incubate the membranes for 1 h at room temperature. Band density was then photographed by the ChemiDOC XRS + system (Biorad) and quantified using the Image Lab software. The detailed antibodies information for Western Blot was listed as follows: CCN1 (Abcam#24448, 1:1000), IL-1b (Cell Signaling Technology#12242S, 1:2000), MPO (Thermo Fisher Scientific#MA1-34067, 1:1000), HSP90 (Cell Signaling Technology#14793, 1:2000), HRP conjugated goat anti-mouse antibody (Biorad#1706516, 1:5000), HRP conjugated goat anti-rabbit secondary (Biorad#1706515, 1:5000).

### Cell culture

Human retinal vascular endothelial cell (HRVEC), 293T, and HL60 cell line were obtained from Otwo Biotech (Shanghai, China) and cultured at 37 °C with 5% CO2. Both HRVEC and 293T were maintained in Dulbecco’s modified Eagle’s medium (DMEM, Corning#10-014-CVR) containing 10% fetal bovine serum (Hyclone#SH30406.05) and 1% penicillin/streptomycin (Hyclone#SV30010-5). HL60 were cultured in RPMI 1640 (Gibco#C11875500CP-10) with L-glutamine (Gibco #25030081) with 25 mM HEPES (HyClone#SH30237.01), 1% penicillin/streptomycin and 10% fetal bovine serum. To differentiate HL60 cells into granulocyte-like cells, the cells were incubated for 5 days with DMSO (1.3%). Cells in passages 3–6 were used to perform experiments.

### Generation of HRVEC^CCN1 OE^ and collection of conditioned media

HRVEC (Passage 4) were seeded in a 6-well plate at a density of 100,000 cells per well. Upon adherence, the DMEM medium was replaced with serum-free Opti-MEM medium, and the cells were starved for 6 h. Subsequently, the Opti-MEM medium was replaced with DMEM medium supplemented with 10 µg/mL polybrene (Polybrene, Sigma-Aldrich, #H8761). Lentivirus overexpressing CCN1 and vector (OBIO Technology, Shanghai, China) were used to infect HRVEC at a multiplicity of infection of 20. Following 48 h of infection, 2 µg/mL puromycin was added to select successfully infected cells. Western blot and flow cytometry were performed to confirm the efficiency of infection. HRVEC at passages 5 or 6 were seeded in a 6-well plate at a density of 100,000 cells per well. 1 mL of fresh medium was added to each well, and the cells were incubated for 12 h before collecting the conditioned media.

### Adherence assay

HRVEC were seeded onto Tissue Culture-Treated 48-Well Plates (Corning#CLS3548) without additional coating and grown until reaching 80% confluency over 48 h. To evaluate the adherence of neutrophils to HRVEC, neutrophils were isolated as described in the method titled “*Human peripheral blood and mouse neutrophil isolation*” and then co-cultured with HRVEC for 4 h. Medium and non-adherent neutrophils were then removed, and adherent cells were washed 3 times with PBS. The number of adherent neutrophils to HRVEC was counted under the microscope after fixation and 0.5% crystal violet staining.

For direct adhesion assays, we harvested conditioned media from HRVEC^Veh^ and HRVEC^CCN1 OE^ cultures and applied them to suspended neutrophils. Before seeding neutrophils, 48-well plates (NEST#748011, untreated surface) were coated with poly-L-lysine (SOLARBIO#YA0170, 50 µg/mL, 100 µL/well) at 37 °C for 1 h. Subsequently, the coating solution was removed, and the plates were washed 3 times with PBS. Neutrophils suspended in conditioned media were seeded into plates and incubated for 4 h. Following the removal of non-adherent cells, the wells were fixed, stained with 0.5% crystal violet, and the adherent cell count was determined under a microscope.

### Transwell chemotaxis assay

HRVEC were seeded on the bottom chamber of a transwell with 3.0 μm Pore Polycarbonate Membrane (Corning, NY, USA) for 48 h. Neutrophils were placed in the top chamber and incubated for 2 h. Neutrophils migrated into the bottom chambers were harvested with PBS containing 5 mM EDTA and their absolute numbers were determined by counter. In the conditioned media-treated experiment, the conditioned media was collected as described in method titled “*Generation of HRVEC*^*CCN1 OE*^*and collection of conditioned media* ” and added into the bottom chamber instead of HRVEC.

### Concentration of supernatant

The medium collected from HRVEC^Veh^ and HRVEC^CCN1 OE^ was concentrated via Amicon® Ultra-2 centrifugal filter devices (MilliporeSigma#1145U09, Darmstadt, Germany) with a molecular weight (MW) cutoff of 50 kDa (MW of CCN1 = 40 kDa), following the manufacturer’s instructions. Briefly, add 2 mL collected medium to the sample chamber of the filter unit. Place the unit in a centrifuge and spin at 4000 g for 1 h at room temperature. After centrifugation, remove the filter unit and collect the filtrate from the upper part of the filter into a collection tube. Invert the tube and centrifuge at 1000 g for 2 min to obtain about 50 µL of concentrated solution. The pre- and post-concentrated and depleted parts were used in our experiment with 1:50 dilution in RPMI-1640 medium for further treatment.

### NETs assay

Neutrophils were plated at 20,000 cells per well in 48-well plates. After incubation in the RPMI 1640 for 1 h, cells were pre-treated with SYTOX Green (Invitrogen#S7020, 1 µM) and then stimulated with rCCN1 (4 ug/mL), ionomycin (4 µM), or an equal volume of PBS for 2.5 h. For experiments that involve CM treatment, CM was added to the wells with 1:50 dilution for 2.5 h. Extracellular DNA release was examined by measuring the green fluorescence with a microplate fluorescence reader (excitation: 485 nm, emission: 527 nm). Neutrophils were then fixed in 4% PFA, followed by Hoechst 33342 (Invitrogen#H3570,1:10,000) staining for 20 min. The quantification of NETs was performed as reported [40]. Briefly, the NETs area was quantified by subtracting the Hoechst 33342 signal from the SYTOX Green signal to remove the nucleus in the quantification.

In immunocytochemistry experiments, the medium was removed, and cells were washed with PBS 3 times after 2.5 h treatment. Cells were then fixed with 4% PFA instantly for 20 min. After fixation, cells were blocked and permeabilized in 10% goat serum and 0.1% triton-X for 1 h at room temperature and then incubated with primary antibodies at 4 °C overnight. Secondary antibodies conjugated fluorescence were used to incubate samples for 1 h at room temperature. Finally, samples were applied with mounting medium with DAPI (Abcam#104139). Slides were photographed under confocal microscopy (Leica TCS SP5, Wetzlar, Germany). Quantification was determined from 3 to 4 non-overlapping fields per sample and the average was used for statistics in every experiment. Both fluorescence intensity and related areas were analyzed with ImageJ software.

### Statistical analysis

Clinical data analyses were performed using R (www.Rproject.org) or Prism (Version 9.5.0, GraphPad Software, LLC.) software, and we considered *P* value < 0.05 to be statistically significant. The Chi-square test or Fisher’s exact test was used for the analysis of categorical variables. Student’s t-test was used for comparing continuous variables. Logistic regression analysis was used to identify significant risk factors for diabetic retinopathy.

## Results

### CCN1 is positively related to the progression of DR

**Fig. 1:**
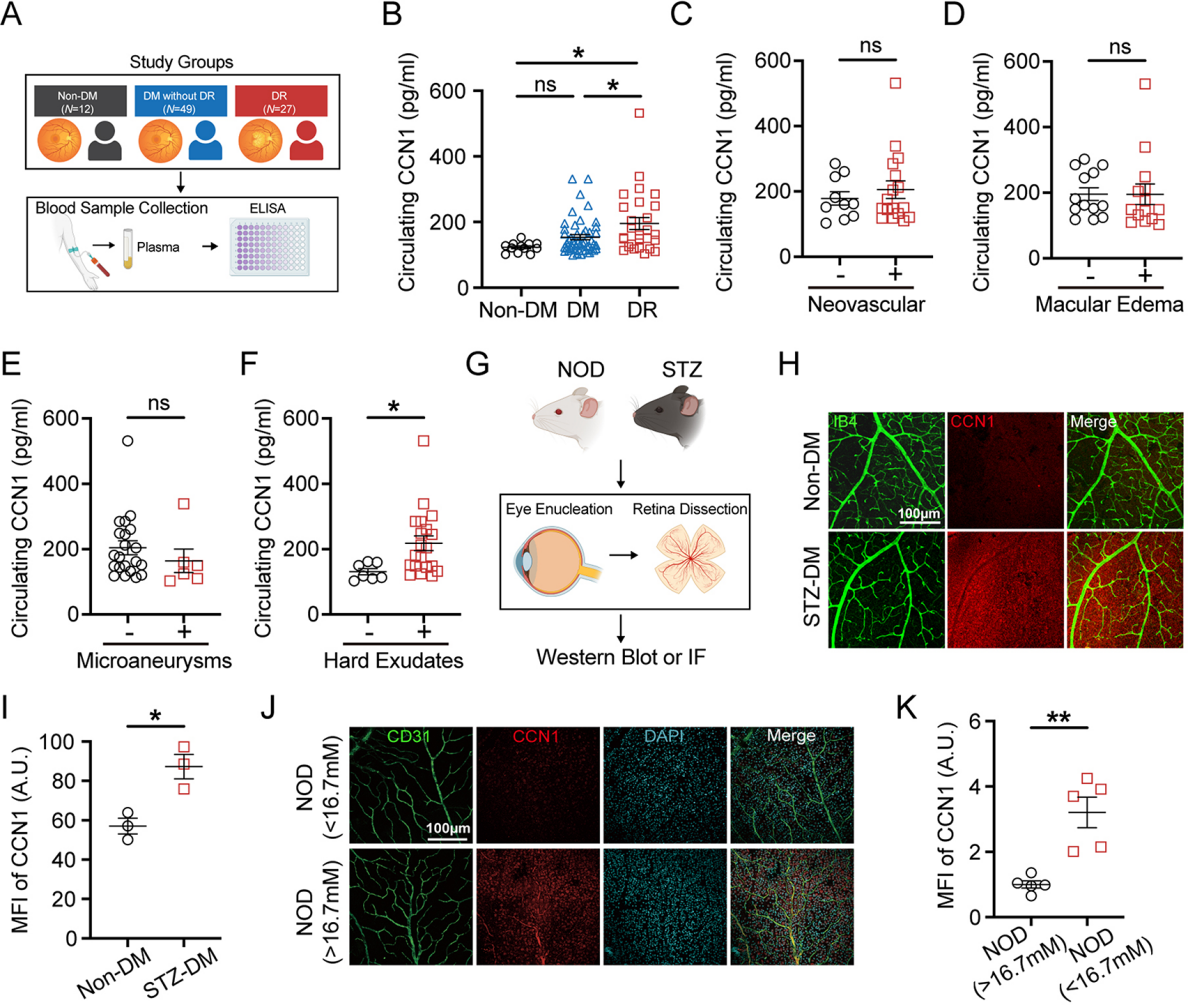
CCN1 is upregulated during the progression of DR. (**A**) Overview of study groups and experimental design. (**B**) Measurement of circulating CCN1 protein levels in peripheral blood across study groups: Non-DM (*N* = 12), DM without DR (*N *= 49), and DR (*N *= 27). **(C-F) **Circulating CCN1 protein level in DR groups with or without neovascular (**C**), macular edema (**D**), microaneurysms (**E**), and hard exudates (**F**). (**G**) Schematic presentation of the diabetic mouse model and retina dissection. (**H-I**) Representative images and mean fluorescence intensity (MFI) quantification of CCN1 and IB4 on retinas of Non-DM and STZ-DM mice (*n* = 3). (**J-K**) Representative images and MFI quantification of CCN1 and CD31 on retinas flat mount of NOD mice with high blood glucose (> 16.7 mM) or normal blood glucose (< 16.7 mM) (*n* = 5). ns: No significance, *: *P* <0.05, **: *P*<0.01. Data are shown as mean ± SEM. Statistical differences were assessed using unpaired, 2-tailed Student’s t-test

To gain insight into the role of CCN1 in DR, we first enrolled participants including control participants without diabetes (Non-DM), diabetes participants without diabetic retinopathy (DM), and diabetes participants with retinopathy complication (DR) to examine the CCN1 protein level change in peripheral blood. 88 participants participated in the study including 12 Non-DM, 49 DM, and 27 DR (Fig. 1A). The mean age of Non-DM, DM, and DR was 54.0, 53.0, and 54.0 years respectively (Table 1). According to the previous report [41], insulin treatment, elevated FBG levels, and higher HbA1c concentration were considered risk factors for a higher prevalence of DR in people with DM. In our study cohort, the percentage of HbA1c and fasting plasma glucose (FBG) rose in diabetic participants as expected (both *P* < 0.001, Table 1), although no significant differences were found between the DR and DM groups. Among these groups, the level of uric acid (UA), estimated glomerular filtration rate (eGFR), Creatinine (Cr) and Serum cystatin C (CysC) was higher in DR than DM (Table S1), indicating poor kidney function in diabetic patients with retinopathy. Medication in DM and DR groups has no statistical difference in our study cohort (Table S2).

**Table 1:** Characteristics of participants by the presence of DM or DR

Variables	Non-DM(*N = 12*)	DM(*N = 49*)	DR(*N = 27*)	*P* overall	*P* ^a^
Sex (Female)	6 (50.0%)	18 (36.7%)	15 (55.6%)	0.262	0.045*
Age (years)	54.0 [49.5;59.2]	53.0 [47.0;59.0]	54.0 [50.5;60.5]	0.750	0.457
BMI (kg/m^2^)	25.6 [23.5;26.6]	25.1 [22.9;26.7]	24.8 [23.1;26.0]	0.530	0.931
Diabetes duration (years)	0.00 [0.00;0.00]	5.00 [0.50;9.00]	11.0 [6.75;17.1]	< 0.001*	< 0.001*
HbA1c (%)	5.80 [5.60;6.40]	8.30 [7.00;10.3]	8.30 [7.20;9.30]	< 0.001*	0.987
FBG (mmol/L)	5.81 [5.14;5.87]	7.86 [6.77;9.17]	8.55 [5.89;10.1]	< 0.001*	0.353
CCN1 (pg/ml)	124 [116;133]	134 [116;169]	160 [128;247]	0.007*	0.036*

*P* values < 0.05 indicates the statistical significance and are shown with an asterisk. Results were presented as mean ± SD for continuous normally distributed variables and or medians (quartile 1, quartile 3) for not normally distributed variables, or n (%) for categorical variables. Abbreviations: BMI: body mass index; FBG: fasting plasma glucose; HbA1c: glycated hemoglobin A1c; CCN1: cellular communication network factor 1; NE: neutrophil elastase.* P*^*a*^: statistical significance between DM and DR group

We then performed an ELISA assay to detect whether the circulating level of CCN1 differed from each group (Fig. 1A). The circulating level of CCN1 in the plasma of DR was higher than that of DM or Non-DM (Fig. 1B). This suggests that increased CCN1 levels are associated with the prevalence of DR. By performing linear correlation analysis of CCN1 and other clinical indicators, we found that CCN1was positively correlated with diabetes durations (Fig. S1A), NLR (Fig. S1B), and neutrophils count (Fig. S1C). To delve further into the relationship between CCN1 and the progression of diabetic retinopathy, we investigated whether CCN1 was involved in the progress of DR. No elevated circulating CCN1 level was observed in DR patients with neovascular (Fig. 1C), macular edema (Fig. 1D) or microaneurysms (Fig. 1E). However, the level of circulating CCN1 in DR patients with hard exudates was higher than those without hard exudates (Fig. 1F), suggesting that CCN1 levels begin to rise in the early stages of DR, and that there is a positive association between CCN1 and active DR. Building on this, we turned our attention to the retinas, the primary site of DR lesions, to investigate whether CCN1 is similarly upregulated in this critical context (Fig. 1G). Our data revealed that CCN1 expression was notably upregulated in the retinas of streptozocin (STZ)-induced diabetic mice (Fig. 1H-I, Fig S1D-E). CCN1 expression was also upregulated in the retinas of NOD mice that had spontaneously progressed to diabetes (random blood glucose levels exceeding 16.7 mmol/L) when compared to their diabetes-free littermates (Fig. 1J-K, Fig S1F-G). Notably, the CCN1 fluorescence is highly co-localized with isolectin B4 (IB4)-positive and CD31-positive endothelial cells (Fig. 1H&J, Fig S1F).

Furthermore, we validated the expression of CCN1 in publicly available single-cell sequencing (scRNA-Seq) data of retinas from various diabetes models. In the retinas of STZ-induced diabetic mice, CCN1 was predominantly expressed in Müller cells and endothelial cells (Fig. S2A). In STZ and high-fat diet (HFD)-treated rats, CCN1 was primarily expressed in endothelial cells (Fig. S2B). It is noteworthy that in both diabetic conditions, there was a discernible trend of increased CCN1 expression, particularly within the endothelial cell population (Fig. S2A-B). Additionally, in scRNA-Seq data from an oxygen-induced retinopathy (OIR) model, which is commonly used to mimic ischemic retinopathies such as retinopathy of prematurity and proliferative diabetic retinopathy [42], we observed CCN1 highly expressed in Müller cells, pericytes, endothelial cells, neural stem cells, and astrocytes (Fig. S2C). Under normoxia conditions, the retinal vasculature of neonatal mice matures within 14 days. As an angiogenic factor, CCN1 was highly expressed at postnatal day 14 (P14) and was rarely detected at P17 in the endothelial cells of retinas in normoxia-exposed mice (NORM) (Fig. S2C). In the OIR model, pathological neovasculature proliferates until P17 and then begins to regress [43]. Intriguingly, the pattern of CCN1 mRNA expression was entirely reversed in OIR retinas; it exhibited a robust upregulation at P17 compared to P14 (Fig. S2C). This result indicated that under physiological conditions CCN1 primarily participates in retinal vasculature development, while under OIR conditions, it may play a role in the regression of pathological vasculature. In summary, our findings indicate that CCN1 is expressed in various cell types during different stages of retinal development but is primarily found in Müller cells and endothelial cells in the mature retinas of mice. Collectively, these results suggest that CCN1 is upregulated in both retinas and peripheral blood under diabetic conditions, and the elevated levels of CCN1 may contribute to the progression of diabetic retinopathy.

### CCN1 promotes retinal leakage

**Fig. 2:**
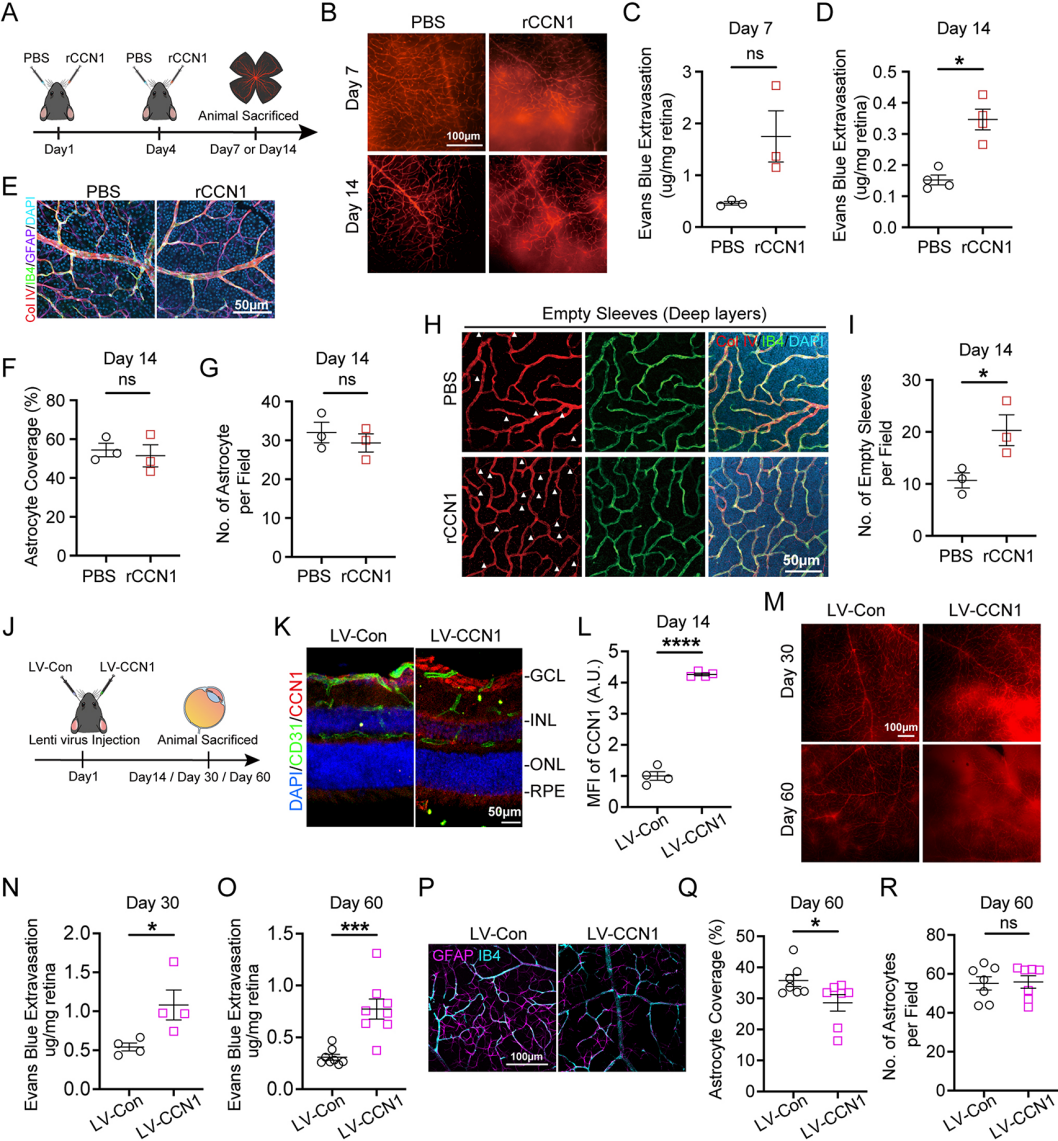
CCN1 induced capillary degeneration and retinal leakage. (**A**) Experimental flow chart for rCCN1 or PBS injection. (**B-D**) Representative images (**B**) and quantification of Evans blue extravasation on day 7 (**C**, *n* = 3) and day 14 (**D**, *n* = 4) after rCCN1 or PBS injection. (**E**) Representative images showing Col IV, CD31, GFAP staining of the rCCN1- or PBS-injected retinas (*n* = 3) on day 14 after rCCN1 or PBS injection. (**F-G**) The percentage of astrocytes’ endfeet coverage around retinal blood vessels (**F**) and the number of astrocytes per field (**G**) on day 14 after rCCN1 or PBS injection. (**H-I**) Representative images (**H**) and the number of empty sleeves in retinal deep layer vessels plexus (**I**, *n* = 3), the white arrows indicate empty sleeves on the image. (**J**) Experimental workflow chart for lentivirus (LV-Con or LV-CCN1) injection. (**K-L**) Representative images of CD31, CCN1 stained retina frozen sections (**K**), and MFI of CCN1 (**L**, *n* = 4) on day 14 post lentivirus injection. (**M-O**) Representative images (**M**) and quantification of Evans blue extravasation on day 30 (**N**, *n* = 4) and day 60 (**O**, *n* = 8). (**P-R**) Representative images of IB4 and GFAP staining on day 60 post lentivirus injection (**P**), the percentage of astrocytes’ endfeet coverage around retinal blood vessels (**Q**, *n* = 7), and the number of astrocytes per field (**R**, *n* = 7). GCL: Ganglion cell layer; INL: Inner nuclear layer; ONL: Outer nuclear layer; RPE: Retinal pigment epithelium. ns: No significance, *: *P* <0.05, ***: *P*<0.001, ****: *P*<0.0001. Data are shown as mean ± SEM

To evaluate the contribution of CCN1 to DR pathogenesis, we then administered rCCN1 directly into the eyes of mice via intravitreal injection (Fig. 2A). Elevated CCN1 within the vitreous cavity led to a trend of increased vascular permeability on day 7 following the first rCCN1 injection but was not statistically significant (Fig. 2B-C). However, by day 14, there was a significant increase in vascular permeability in the retinas of mice treated with rCCN1 (Fig. 2D). To further explore the impact of CCN1 on retinal vasculature, we examined various vessel parameters. Interestingly, rCCN1 injection did not result in changes to the vessel percentage, total vessel length, or the number of junctions or endpoints of the retinal vasculature (Fig. S3A, B-E). Astrocytes play a crucial role in BRB, and their support is essential for maintaining retinal vascular integrity. Our data revealed that the number of astrocytes per field and the extent of astrocyte coverage were not significantly altered by the rCCN1 treatment (Fig. 2E-G). Of particular interest, we investigated non-functional empty basement membrane sleeves, which are acellular and express collagen IV but not IB4 and are associated with limited flow [44]. An increased number of these acellular empty sleeves suggests heightened capillary degeneration, which is a significant contributor to the progression of DR [45]. Strikingly, we observed an elevated number of acellular empty sleeves in the deep vessel plexus (Fig. 2H-I), but not in the intermediate vessel plexus (Fig. S3F-G), of the retinas that had been injected with rCCN1 on day 14, suggesting the role of CCN1 in promoting capillary degeneration.

**Fig. 3:**
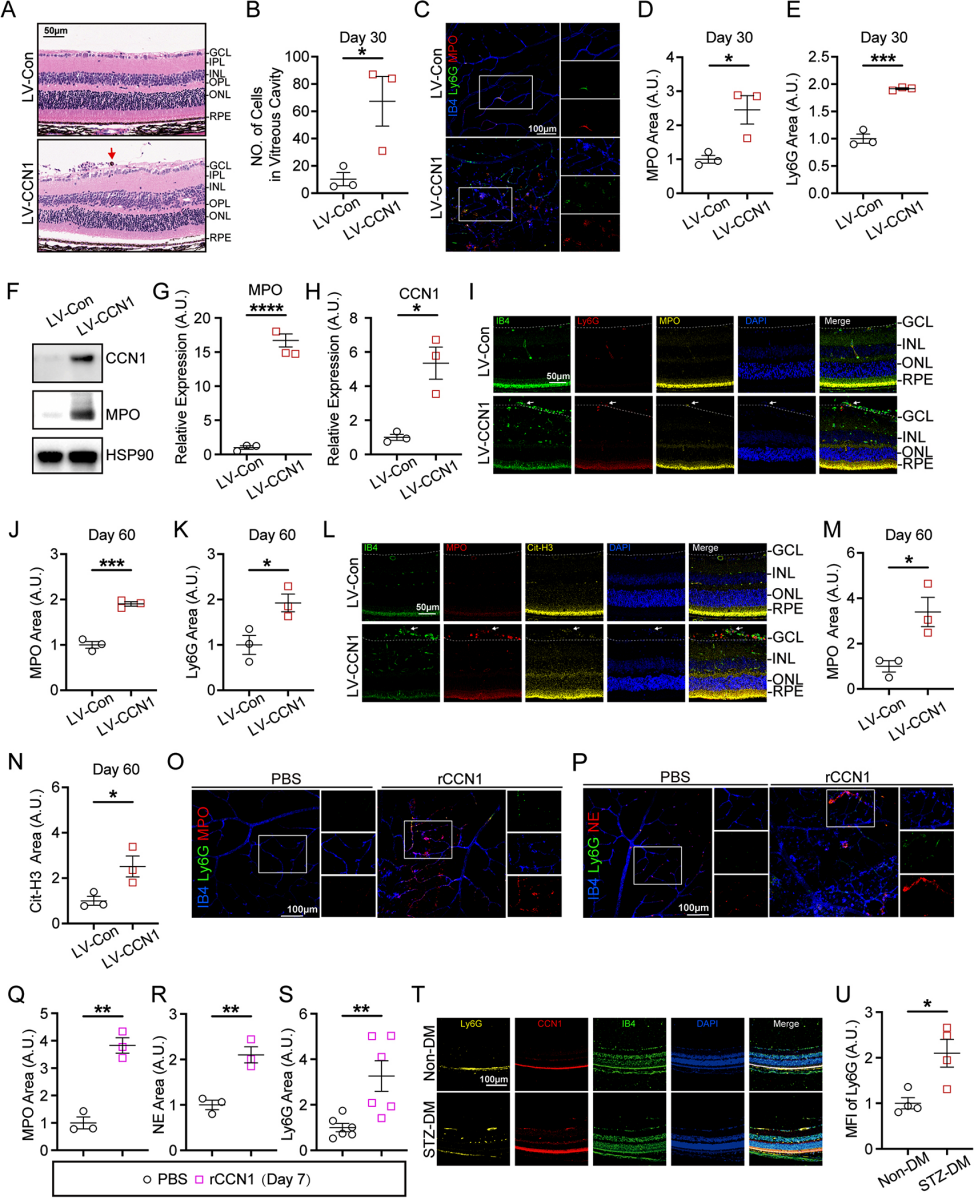
CCN1 induces NETs extrusion in mouse retina. (**A-B**) Representative hematoxylin and eosin (H&E) staining of the retina (**A**) and the number of cells in the vitreous cavity per section (**B**) day 30 post lentivirus injection (*n* = 3). (**C-E**) Representative images of IB4, Ly6G, MPO staining in retina flat mount day 30 post lentivirus injection and positive fluorescence area quantification of MPO (**D**) and Ly6G (**E**), *n* = 3. (**F-H**) Representative immunoblots (**F**) and densitometry quantification of MPO (**G**) and CCN1 (**H**) of retina tissue dissected on day 30 post lentivirus injection, *n* = 3. (**I-K**) Representative images of IB4, Ly6G, MPO staining on retina frozen section day 60 post lentivirus injection and positive fluorescence area quantification of MPO (**J**) and Ly6G (**K**), *n* = 3. (**L-N**) Representative images of IB4, Cit-H3, MPO staining in retina frozen section day 60 post lentivirus injection and positive fluorescence area quantification of MPO (**M**) and Cit-H3 (**N**), *n* = 3. (**O-S**) Representative images of IB4, Ly6G, MPO staining (**O**) and IB4, Ly6G, NE staining (**P**) on retina flat mount day 7 post rCCN1 injection and positive fluorescence area quantification of MPO (**Q**, *n* = 3), NE (**R**, *n* = 3) and Ly6G (**S**, *n* = 6). (**T**-**U**) Representative images of IB4, Ly6G, CCN1 staining in retina frozen section of STZ-DM mice retinas (**T**) and MFI of Ly6G (**U**, *n* = 4). IPL: Inner plexiform layer; OPL: Outer plexiform layer. *: *P* <0.05, **: *P*<0.01, ***: *P*<0.001, ****: *P*<0.0001. Data are shown as mean ± SEM. Statistical differences were examined by unpaired, 2-tailed Student’s t-test in data

Considering the limited duration of recombinant protein administration, we established a model of continuous CCN1 overexpression within the eye by intravitreal injection of lentivirus (Fig. 2J). The successful overexpression of CCN1 was confirmed through immunofluorescent staining (Fig. 2K-L, S3H-I) and western blot analysis (Fig. 3F, H) on day 14. Our findings revealed a significant increase in vascular permeability in the retinas with CCN1 overexpression on day 30, and this effect was sustained through day 60 (Fig. 2M-O). Importantly, the vessel percentage, total vessel length, and the number of junctions or endpoints of the retinal vasculature remained unaltered following lentivirus-mediated CCN1 overexpression (Fig. S3I-M). However, we observed a reduction in astrocyte coverage after CCN1 overexpression (Fig. 2P-Q), even though the number of astrocytes remained unchanged (Fig. 2R). These results collectively support the role of CCN1 in promoting retinal leakage through the induction of capillary degeneration.

### CCN1 boosts neutrophil stasis and NETs extrusion

Our previous findings have shown that elevated levels of CCN1 in the retina resulted in capillary degeneration and increased retinal leakage. To further elucidate the role of CCN1 in DR, we sought to determine whether CCN1 overexpression also leads to retinal neurodegeneration. However, CCN1 overexpression did not alter the number of neuron cells or the thickness of retinal layers (Fig. S4F-H). This suggests that the increase in CCN1 primarily contributes to the augmentation of retinal leakage rather than neuronal degeneration in DR. Microvessels, especially capillaries, are the sites most susceptible to neutrophil stasis and represent the severest sites of leakage in the progression of DR [46]. Therefore, we proceeded to investigate whether the overexpression of CCN1 in retinas leads to an upregulation of neutrophil stasis within capillaries. Upon examination of the entire eye section, we observed a significant accumulation of cells, including neutrophils, in the vitreous cavity (Fig. 3A-B). Furthermore, aggregated neutrophils were observed at the branch points of vessels, and NETs appeared around vessels on day 30 following LV-CCN1 injection, as indicated by the increased MPO and Ly6G positive area on retina flat-mount (Fig. 3C-E). Consistently, MPO levels were markedly increased in parallel with the elevation of CCN1 throughout the whole retina (Fig. 3F-H). To gain further insight into the location of NETs within the retina, we performed immunofluorescent staining of retinal frozen sections on day 60 after injection. Our observations revealed an accumulation of immune cells on the surface of the inner retina, and we noted that NETs were present on retinas overexpressed CCN1, as indicated by the colocalization of MPO and Ly6G (Fig. 3I-K) or MPO and Cit-H3 (Fig. 3L-N). This suggests that CCN1 modulates the adhesion, migration, and NETs extrusion by neutrophils. In accordance with CCN1 overexpression by lentivirus, the supplementation of rCCN1 also led to neutrophil stasis and NETs formation on day 7, characterized by the colocalization of MPO and Ly6G (Fig. 3O, Q, S) or MPO and NE (Fig. 3P, R, S). However, these effects diminished on day 14 (Fig. S4D-E). Remarkably, despite the reduction in NETs, the vascular permeability on day 14 was significantly increased (Fig. 2D). As previously reported, NETs disrupt endothelial integrity and cause vascular leakage [15, 47]. Hence, the abnormal neutrophil stasis and NETs in the retina appear to be resolved over time, while the disruption of retinal vasculature can persist for a longer duration. In the retinas of STZ-DM mice, more Ly6G^+^ fluorescence was evident and colocalized with CCN1 and IB4 (Fig. 3T-U), suggesting increasing neutrophil adherence to retinal vessels under diabetic conditions. In summary, these findings collectively support the role of CCN1 in modulating neutrophil stasis and the NETs extrusion in the context of DR.

Neutrophil stasis and subsequent inflammation are significant contributors to the pathogenesis of DR, and this complex process involves various cell types, including microglia, Müller cells, and leukocytes [48]. In our study, we observed a substantial increase in the disorganized IB4^+^ area in retinas treated with rCCN1 supplementation (Fig. S4A). This area extended from the inner retina to the outer retina, in contrast to the control retinas where the IB4^+^ area was primarily confined to the inner retina (Fig. S4A). It’s worth noting that while IB4 is often used as a vascular indicator, it can also be utilized to label microglia and macrophages [49]. Furthermore, microglial cells predominantly reside in the inner plexiform layer (IPL) and outer plexiform layer (OPL) of the retina and remain in a quiescent or resting state under normal conditions. However, they become activated and migrate toward the outer retina in response to diabetic conditions [50, 51]. Hence, rCCN1 supplementation may trigger an inflammatory cascade and gliosis in the retina, as confirmed by the co-staining of IB4 and CD31 (Fig. S4B-C). This observation was further corroborated by co-staining with IBA1, a marker for microglia cells in the retinas (Fig. S4I). Consistent with the activation of microglia cells in rCCN1-injected retinas, we also observed an increase in both IB4^+^ and IBA1^+^ areas in the retinas injected with LV-CCN1 (Fig. S4I-K). On day 60 after LV-CCN1 injection, the IBA1^+^ area was notably expanded in the IPL and OPL (Fig. S4I), indicating that the elevated CCN1 in the retina induced both NETs extrusion and microglial cell activation. In summary, our findings suggest that CCN1 is also a catalyst for microglial cell activation in retinas.

### CCN1 enhances the adhesion and migration of neutrophils

**Fig. 4:**
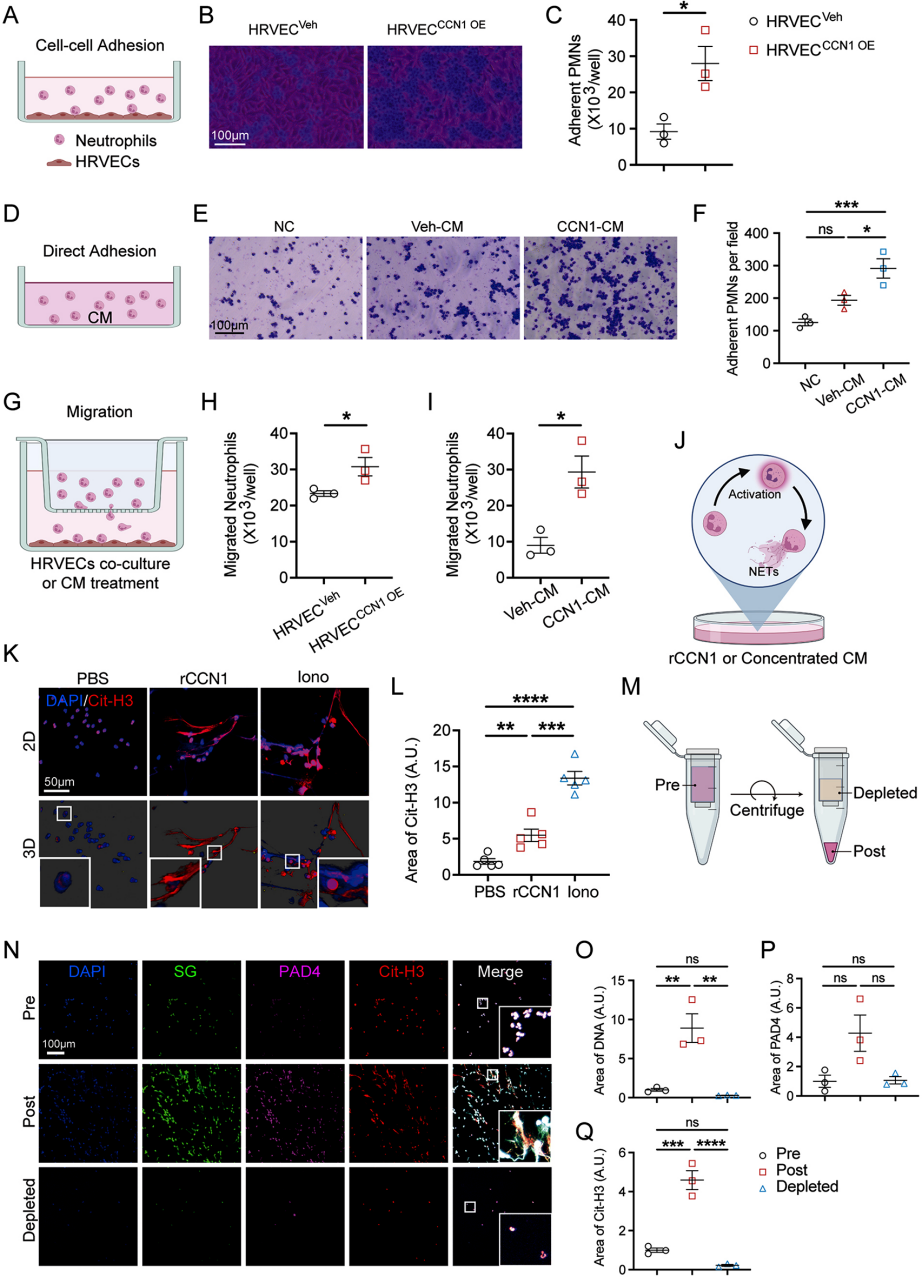
CCN1 mediates adherence, migration, and NETs extrusion of neutrophils *in vivo.* (**A**) Schematic diagram of co-culture of neutrophils with HRVEC overexpressed CCN1 (HRVEC^CCN1 OE^) or vehicle (HRVEC^Veh^). (**B-C**) Visual assessment of human primary neutrophil adherence to HRVEC after 4-h co-culture (**B**) and quantification of the number of adherent neutrophils per field (**C**), assessed by crystal violet staining (adherent neutrophils appear dark purple) (*n* = 3). (**D**) Schematic diagram of co-culture of neutrophils with conditioned media (CM) derived from HRVEC^CCN1 OE^ or HRVEC^Veh^. (**E-F**) Visual assessment of human primary neutrophil adherence to the plate after 4-h co-culture with CM and the quantification of the number of adherent neutrophils per field (**E**), assessed by crystal violet staining (**F**, *n* = 3). (**G**) Schematic diagram of co-culture of human primary neutrophils with HRVEC or CM in transwell. (**H-I**) The number of migrated neutrophils in the lower chamber with HRVEC co-culture (**H**, *n* = 3) and CM treatment (**I**, *n* = 3) for 2 h. (**J**) Schematic diagram of treating human primary neutrophils with rCCN1 or concentrated CM. (**K-L**) Representative images and 3D construction of human primary neutrophils stained with Cit-H3 and DNA (DAPI) (**K**), and quantification of Cit-H3^+^ area (**L**, *n* = 4). (**M**) Schematic diagram of the CM concentration process; highlighting the pre-concentrated media, post-concentrated media, and the depleted part were preserved for experiments. (**N-Q**) Representative images of human primary neutrophils treated with pre-, post-, and depleted CM for 2.5 h (**N**), and quantification of DNA^+^ (**O**), PAD4^+^ (**P**), Cit-H3^+^ (**Q**) area (*n* = 3). ns: no significance, *: *P* <0.05, **: *P*<0.01, ***: *P*<0.001, ****: *P*<0.0001. Data are shown as mean ± SEM. Statistical differences were examined by unpaired, 2-tailed Student’s t-test

To further explore the potential involvement of CCN1 in modulating neutrophil behavior, we delved into the underlying mechanisms of how CCN1 interacts with neutrophils in vitro. We first established an HRVEC cell line overexpressing CCN1 (HRVEC^CCN1 OE^) and isolated human primary neutrophils from whole peripheral blood for adhesion assay (Fig. 4A). Our cell-cell adhesion assay revealed that a greater number of neutrophils adhered to HRVEC^CCN1 OE^ when compared to HRVEC^Veh^ (Fig. 4B-C), indicating that CCN1 mediated neutrophils-to-endothelial cell adhesion. Beyond its role in modulating cell-cell adhesion, we found that pre-treatment with a conditioned media enriched with CCN1 (CCN1-CM) increased the number of adherent neutrophils (Fig. 4D-F) and dHL60s (Fig. S5A-B), which indicated that CCN1 also directly supported neutrophils-matrix adhesion. To assess the impact of CCN1 on neutrophil migration, we conducted migration assays (Fig. 4G). In both co-culture with HRVEC^CCN1 OE^ and treatment with CCN1-CM, the number of migrated neutrophils exhibited a significant increase compared to the control conditions (Fig. 4H-I). Therefore, the above results collectively suggest that CCN1 promotes the adhesion and migration of neutrophils and potentially captures neutrophils within retinas.

To further confirm that CCN1 contributes to NETs extrusion, we utilized rCCN1 and concentrated conditioned media to treat human primary neutrophils and mouse bone marrow neutrophils (Fig. 4J) in vitro. Under rCCN1 treatment, NETs was detected in both human (Fig. S5C-E) and mouse neutrophils (Fig. S5F) indicated by increased SYTOX Green fluorescence or area. Moreover, co-staining of DNA and Cit-H3 further confirmed that CCN1 contributed to NETs extrusion (Fig. 4K-L, S5H-J). Additionally, ROS production was induced by rCCN1 treatment in both human (Fig. S5G) and mouse neutrophils (Fig. S5K). This further supports the idea that CCN1 activates neutrophils and leads to NETs extrusion. Furthermore, concentrated CCN1-CM also strongly induced NETs (Fig. 4N-Q). In conclusion, our experimental evidence underscores the role of CCN1 in modulating neutrophil adherence, migration, and NETs extrusion.

### Suppressing CCN1 attenuates retinal leakage in diabetic mice

**Fig. 5:**
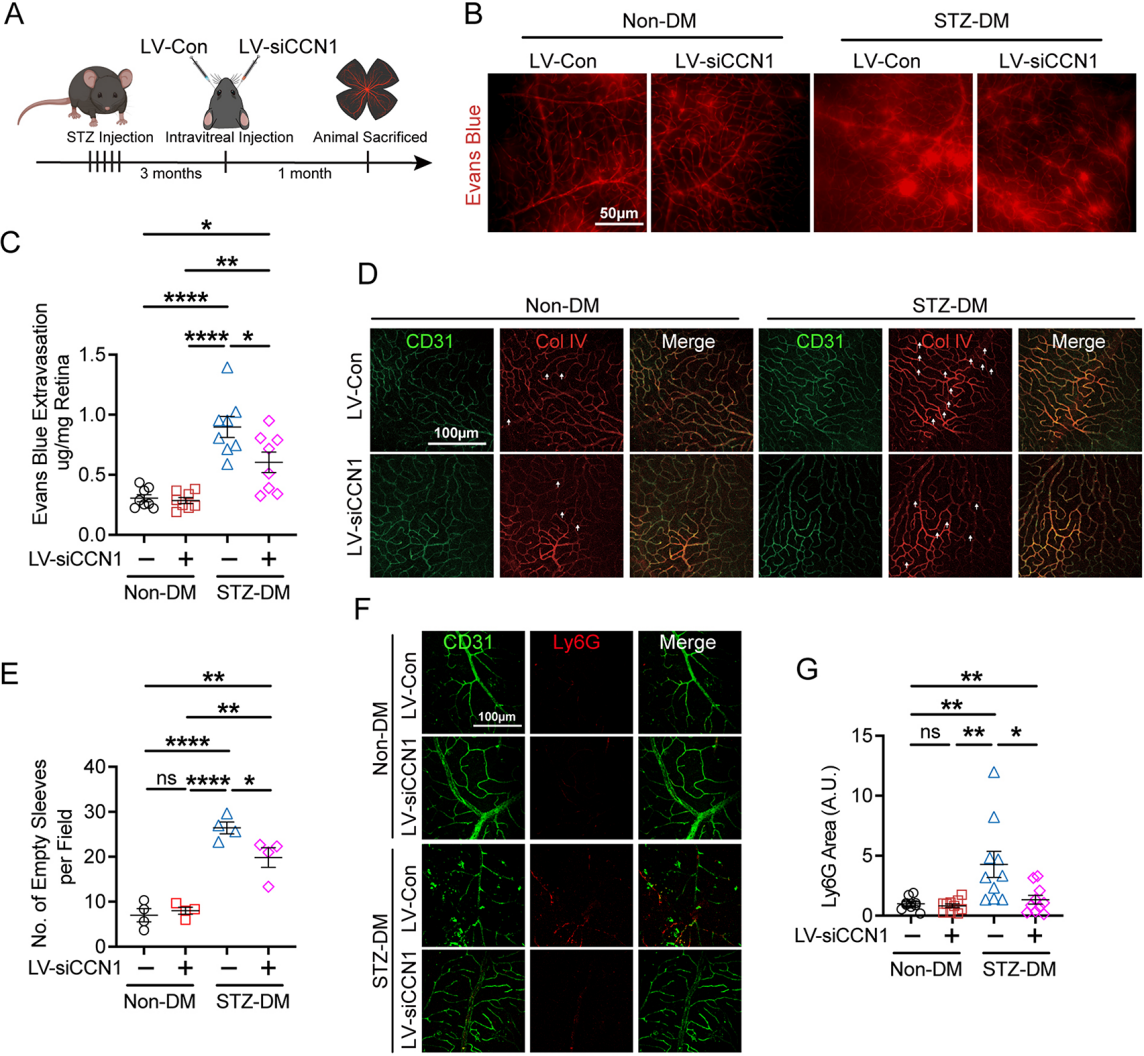
Knockdown of CCN1 reduces NETs extrusion and retinal leakage. (**A**) Schematic of lentivirus injection in diabetic mouse experiment workflow. (**B-C**) Representative retinal flat mount images with Evans blue dye (**B**) and quantification of Evans blue extravasation per retina 1 month after intravitreal LV-siCCN1 or LV-Con injection in STZ-DM or Non-DM mice (**C**, *n* = 8). (**D-E**) Representative retinal flat mount images stained with CD31 and Col IV (**D**) and the number of empty sleeves (**E**, *n* = 4) in retinal deep layer vessels plexus, the white arrow indicates one classical empty sleeve on the image. (**F-G**) Representative retinal flat mount images stained with CD31 and Ly6G (**F**) and the quantification of Ly6G^+^ area (**G**, *n* = 10). ns: No significance, *: *P*<0.05, **: *P*<0.01, ****: *P*<0.0001. Data are shown as mean ± SEM. Statistical differences were examined by unpaired, 2-tailed Student’s t-test

Based on the accumulating evidence, CCN1 might increase retinal leakage by promoting neutrophil stasis and triggering NETs extrusion. Therefore, targeting CCN1 offers a potential strategy to alleviate retinal leakage under diabetic conditions. To further evaluate whether reducing CCN1 could diminish neutrophil stasis and consequently ameliorate retinal leakage, we conducted intravitreal injections to knock down CCN1 expression in STZ-DM mice (Fig. 5A). It’s worth noting that the intravitreal injection did not impact blood glucose levels (Fig. S6A). The LV-siCCN1 treatment led to a significant decrease in CCN1 expression in the retinas of STZ-DM mice (Fig. S6B-C). Consistent with CCN1 reduction, LV-siCCN1 effectively mitigated retinal leakage in STZ-DM mice (Fig. 5B-C). However, LV-siCCN1 did not elicit alterations in the vasculature parameters (Fig. S6D-H) and astrocyte coverage (Fig. S6I-J). We also observed a significant increase in pathological acellular empty sleeves in the retinas of STZ-DM mice, which declined after CCN1 knockdown (Fig. 5D-E). As expected, CCN1 knockdown also resulted in a reduction of neutrophil stasis (Fig. 5F-G), strongly suggesting that CCN1 is responsible for neutrophil stasis, capillary degeneration, and retinal leakage in DR. This finding further underscores the pivotal role of CCN1 in facilitating the progression of DR.

### DNase I alleviates CCN1-dependent retinal leakage

**Fig. 6:**
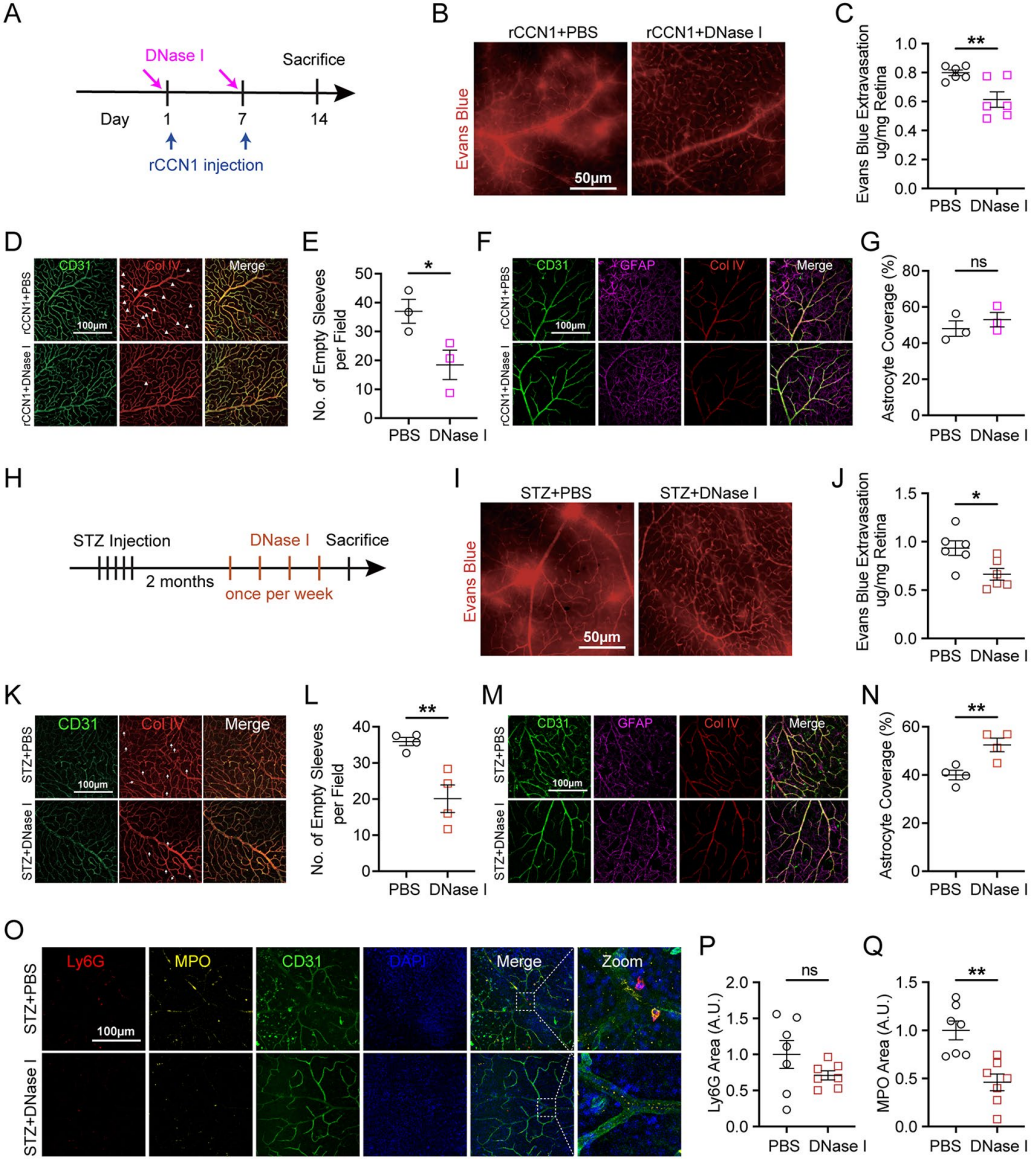
Clearance of NETs with DNase I alleviated retinal leakage. (**A**) DNase I and rCCN1 treatment workflow schematic. (**B-C**) Representative retinal flat mount images with Evans blue dye (**B**) and quantification of Evans blue extravasation per retina (**C**, *n* = 6) one week after the second DNase I injection. (**D-E**) Representative retinal flat mount images stained with CD31 and Col IV (**D**) and the number of empty sleeves (**E**, *n* = 3) in retinal deep layer vessels plexus, the white arrow indicates classical empty sleeve on the image. (**F-G**) Representative images of CD31, GFAP, and Col IV on retina flat mount (**F**) and the percentage of astrocytes’ endfeet coverage around retinal blood vessels (**G**, *n* = 4). (**H**) Schematic of DNase I treatment workflow on STZ-DM or Non-DM mice. (**I-J**) Representative retinal flat mount images with Evans blue dye (**I**) and quantification of Evans blue extravasation per retina one week after last injection (**J**, *n* = 6). (**K-L**) Representative retinal flat mount images stained with CD31 and Col IV (**K**) and the number of empty sleeves (**L**, *n* = 4) in retinal deep layer vessels plexus, the white arrow indicates one classical empty sleeve on the image. (**M-N**) Representative images of CD31 and GFAP on retina flat mount (**M**) and the percentage of astrocytes’ endfeet coverage around retinal blood vessels (**N**, *n* = 4). (**O-Q**) Representative retinal flat mount images stained with MPO, Ly6G, and CD31 (**O**) and the quantification of Ly6G^+^ area (P) and MPO^+^ area (**Q**, *n* = 7). ns: No significance, *: *P* <0.05, **: *P*<0.01. Data are shown as mean ± SEM. Statistical differences were examined by unpaired, 2-tailed Student’s t-test

To further investigate the contribution of NETs in CCN1-dependent retinal leakage, we injected DNase I into the vitreous humor 30 min before rCCN1 injection to determine if DNase I pretreatment would abrogate the negative effects caused by rCCN1 (Fig. 6A). As a result, DNase I pretreatment significantly improved retinal leakage (Fig. 6B-C) and reduced the number of empty sleeves (Fig. 6D-E), although it did not affect astrocyte coverage (Fig. 6F-G). We further explored the effects of DNase I treatment in diabetic retinas to observe whether DNase I could improve retinal leakage (Fig. 6H). Consequently, the inhibition of NETs through DNase I significantly ameliorated retinal leakage (Fig. 6I-J) and decreased the number of empty sleeves (Fig. 6K-L). Interestingly, DNase I treatment also significantly increased astrocyte coverage in the retinas of STZ-DM mice (Fig. 6M-N). Notably, DNase I treatment promoted the degradation of NETs and ameliorated retinal inflammation (Fig. 6O-Q). In summary, these comprehensive findings suggest that CCN1 induces retinal leakage by promoting neutrophil stasis within the retinal microvasculature and modulating NETs extrusion. By suppressing CCN1 or abolishing NETs, retinal leakage can be significantly reduced, providing a novel insight into the treatment of diabetic retinopathy.

### Circulating CCN1 and NE are potentially valuable markers for the assessment of DR

**Fig. 7:**
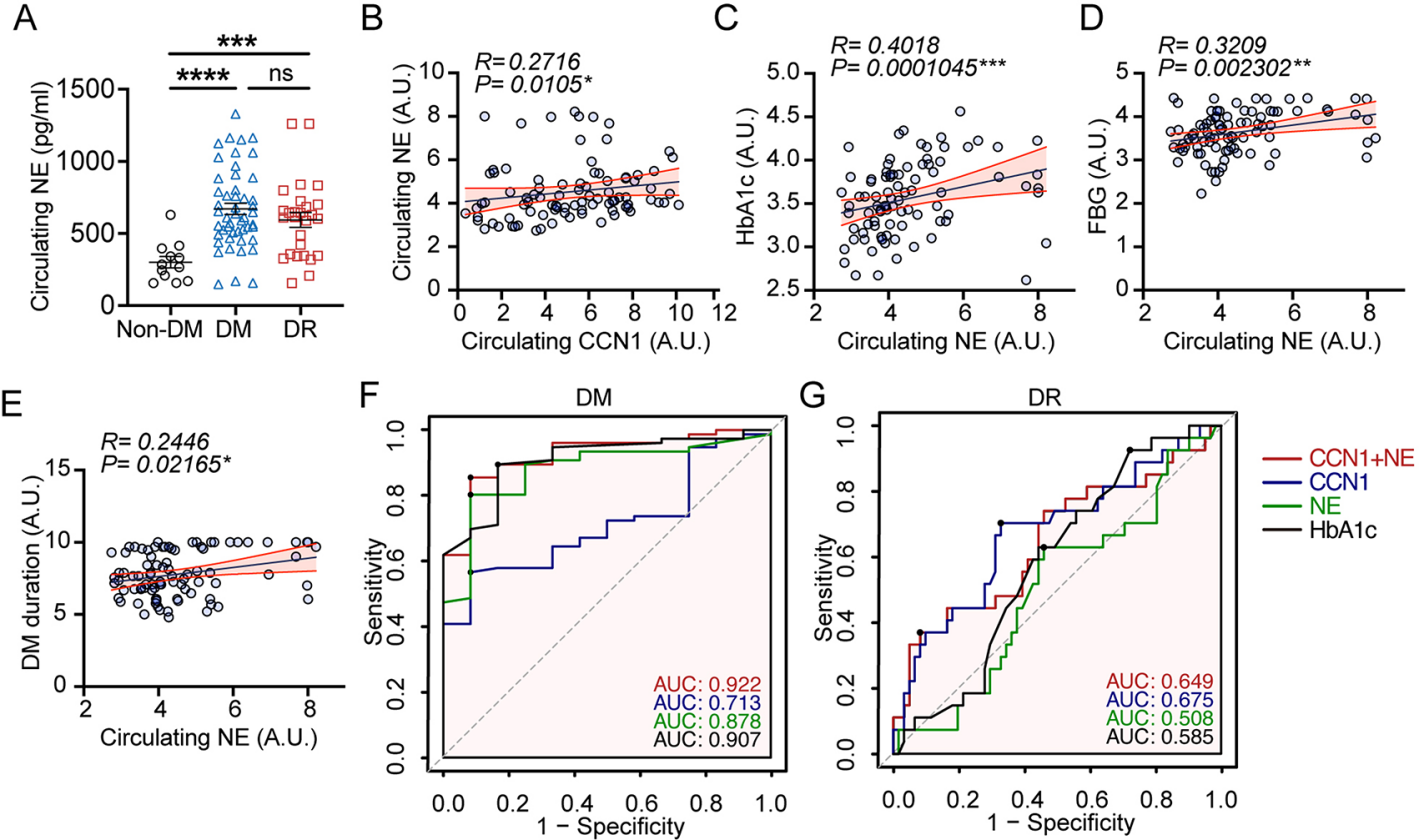
Circulating CCN1 and NE are potentially valuable markers for the assessment of DR. (**A**) Circulating NE protein level in peripheral blood of Non-DM (*N *= 12), DM (*N *= 49), and DR (*N *= 27). (**B-D**) Linear correlation among circulating CCN1 and circulating NE (**B**), HbA1c and NE (**C**), FBG and NE (**D**), DM duration and NE (**E**) in the whole study population (*N *= 88). (**F**) ROC analysis was performed to evaluate the performance of CCN1 + NE, CCN1, NE, and HbA1c in distinguishing DM (including DR) from Non-DM (Non-DM, *N *= 12; DM, *N *= 76). (**G**) ROC analysis was performed to evaluate the performance of CCN1 + NE, CCN1, NE, and HbA1c in distinguishing DR from DM (DM, *N *= 49; DR, *N *= 27). ns: No significance, ***: *P*<0.001, ****: *P*<0.0001. The Spearman correlation test was conducted in data **B-E**. Data are shown as mean ± SEM. Statistical differences were examined by unpaired, 2-tailed Student’s t-test

**Fig. 8:**
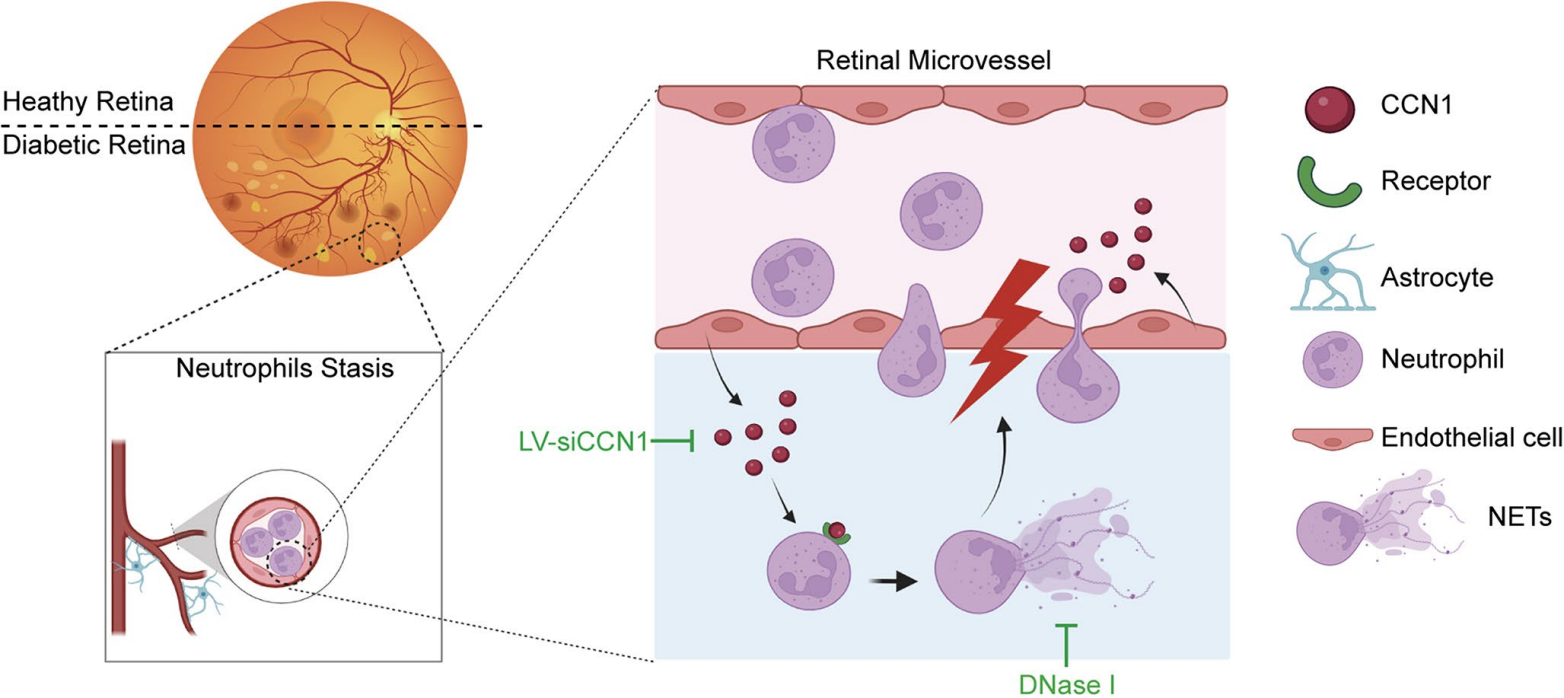
Schematic outlining the process of CCN1 attracting neutrophils and inducing NETs in diabetic retinas. Under diabetic conditions, there is an upregulation of CCN1 within the retinal microenvironment, fostering heightened attraction and subsequent accumulation of neutrophils within retinal vessels. Consequently, neutrophils adhere to the endothelium and undergo migration beyond the vascular barrier. Augmented levels of CCN1 secretion activate neutrophils, precipitating the extrusion of NETs, thereby contributing to the progression of DR. The illustrative depiction was generated using the Biorender website (https://app.biorender.com)

Finally, we investigated the circulating level of NE in our study cohort. Our findings indicated that the circulating level of NE was increased in both DM and DR patients when compared to Non-DM (Fig. 7A). However, there were no significant differences between DR and DM patients. Nevertheless, NE was found to be positively correlated with CCN1 (Fig. 7B). Circulating NE was positively correlated with CCN1, HbA1c, FBG, DM duration, and TG (Fig. 7B-E, Fig S7A), thereby supporting the potential role of NE in the pathology of diabetes. Consequently, we constructed receiver operating characteristic (ROC) curves to determine whether circulating CCN1 or NE could better predict the presence of DR and DM. As illustrated in Fig. 7F, the area under the curve (AUC) of a combination of CCN1 and NE was higher than that of HbA1c, suggesting that combining CCN1 and NE can effectively identify the presence of DM. To predict DR, CCN1 outperformed NE and HbA1c (Fig. 7G). In conclusion, these findings lead us to conclude that CCN1 is intricately involved in the pathogenesis of DR, and it may play a role in modulating the physiological behavior of circulating neutrophils, making it a potentially valuable marker for the assessment of DR (Fig. 8).

## Discussion

Initially, CCN1 was primarily characterized as an angiogenesis factor [17] and was found to exert influence over pathological angiogenesis in patients with PDR [18, 20] and within the retinas of diabetic animal models [22, 52, 53]. Furthermore, previous research had established a positive correlation between CCN1 levels and the extent of neutrophil infiltration within lesion tissues [54]. This observation prompted us to embark on an exploration of the potential link between CCN1 and neutrophils. In our previous study, we unveiled the capacity of CCN1 to induce injury in endothelial cells [21]. In this present investigation, we not only observed an elevation in CCN1 levels in the peripheral blood of patients with DR and in the retinas of diabetic mice, but provided conclusive evidence demonstrating that CCN1 instigated the adhesion, migration, and NETs extrusion of neutrophils. Additionally, treatment with LV-siCCN1 or DNase I yields significant amelioration of neutrophil adhesion to the endothelial lining, consequently reversing the diabetes-induced retinal permeability. Collectively, these findings unequivocally validate the pivotal role of CCN1 in shaping the biological behavior of neutrophils, expanding its functional repertoire beyond its previously reported functions. More importantly, it stands to reason that the targeting of CCN1 may hold considerable promise in curtailing neutrophil stasis and, by extension, halting the progression of DR.

The influence of CCN1 on BRB disruption is discernible across all the constituent cell types comprising the BRB. Firstly, CCN1 exhibits the capacity to incite sterile inflammation, even in the absence of infection [24], therefore causing endothelial cell injury. Secondly, CCN1 triggers pericyte apoptosis, exacerbating the compromise of BRB integrity [55]. Thirdly, our investigation reveals that CCN1 overexpression corresponds to a reduction in astrocyte coverage. Lastly, we observe that CCN1 overexpression induces gliosis and activates microglial cells. Contrary to our findings, Lulu Yan et al. have reported that the loss of CCN1 led to microglial cell activation in the retinas of the OIR model [56]. Given that microglial cells function as the resident macrophages of the retina and are responsive to inflammatory cues [57], we posit that the activation of microglial cells detected in our study is more likely attributed to inflammation rather than a direct consequence of CCN1. Furthermore, the dynamic nature of CCN1 expression in various cell types within the retina, as previously described, may account for the divergent conclusions stemming from its differential roles at distinct life stages. The elevation of CCN1 in the postnatal retina appears to play a pivotal role in maintaining retinal homeostasis during vasculature development. However, within the context of the adult retina under diabetic conditions, CCN1 assumes a contradictory role. Last but not least, despite the anticipated damaging effects of a pronounced inflammatory cascade upon neurons [58], our study does not reveal any significant alterations in neuron numbers following CCN1 treatment. This intriguing phenomenon may be attributable to CCN1’s multifunctionality, as it has been reported to exhibit neuroprotective properties within retinal tissue [59]. Consequently, the role of CCN1 in DR is indisputably multifaceted, rather than singular. CCN1 can be likened to a double-edged sword, capable of inflicting catastrophe upon the BRB while simultaneously conferring neuroprotective benefits.

Our data demonstrated a noteworthy correlation between CCN1 and the duration of diabetes, in keeping with findings from the study by Bin Feng et al. [60] and Zhao-Yu Xiang et al. [61]. Notably, we have provided new and compelling evidence, showcasing elevated levels of circulating CCN1 in DR patients presenting with hard exudates. This newfound insight further substantiates the role of CCN1 in promoting retinal leakage, shedding light on its multifaceted impact on DR progression. Additionally, a multitude of studies have consistently demonstrated the elevation of circulating NETs markers in DR patients when compared to diabetic patients without DR [8, 62, 63]. Among these markers, circulating NE has been identified as an independent risk factor of DR [7]. However, no significant variance was detected between the DM and DR groups in our study. This observation may be attributed, in part, to the relatively small number of participants in our study. Additionally, it is worth noting that elevated circulating NE is not exclusive to retinopathy complications; it is also observed in other microvascular and macrovascular complications, such as atherosclerosis, nephropathy, and neuropathy [64]. Importantly, we did not exclude patients with these complications in the DM group, potentially contributing to the higher levels of circulating NE. Moreover, our findings reveal a positive correlation between circulating NE and diabetes duration, FBG, and HbA1c, in line with previous research [62]. Given the associations of circulating NE with multiple clinical indicators and its link to various diabetic complications, the absence of a difference in NE levels between the DR and DM groups may, at least in part, be attributed to these confounding factors. Consequently, circulating CCN1, rather than NE, emerges as a potential risk factor and biomarker for DR. However, it is noteworthy that our results highlight that the combination of circulating CCN1 and NE yields higher diagnostic accuracy for DM than HbA1c. This suggests that the combined assessment of circulating CCN1 and NE may also serve as a risk factor and biomarker for DM.

However, the precise mechanism by which CCN1 activates neutrophils and triggers NETs extrusion remained unelucidated in our present study. The mechanisms of NETosis (the process of NETs extrusion) are still controversial and warrant further investigation. To date, three distinct mechanisms have been identified in NETosis, categorized as suicidal NETosis, vital NETosis, and mitochondrial NETosis [65]. Suicidal NETosis is dependent on NOX and culminates in neutrophil death [66]. In contrast, vital NETosis and mitochondrial NETosis are believed to result in NETs release without causing neutrophil death [67, 68]. In DR, NETs triggered by hyperglycemia are dependent on NOX and ROS production, signifying that neutrophils confined within the retinal microvasculature undergo suicidal NETosis [8]. Additionally, activated endothelial cells can enhance NETs formation by releasing cytokines like IL-1β and ROS, while NETs can induce endothelial cell activation through protease components like histones, creating a positive feedback loop [69, 70]. Notably, our prior research has revealed that CCN1 promotes NOX4 activation and increases ROS production in human retinal vascular endothelial cells [21]. In this study, heightened ROS production was also observed in neutrophils after rCCN1 treatment. Therefore, CCN1 may induce suicidal NETosis by activating the NOX/ROS signaling pathway. As a secreted protein, CCN1 initiates intracellular signaling pathways through interactions with cell membrane receptors, such as integrins [24, 65]. Furthermore, it has been reported that CCN1 directly binds to Toll-like receptors to activate neutrophil mobilization [24]. Hence, CCN1 may potentially stimulate NETs extrusion in a NOX-dependent manner via interaction with surface receptors on neutrophils, although this hypothesis necessitates further validation. Moreover, prior research has recognized CCN1 as a mediator of leukocyte migration and vascular inflammation [28]. Our data reinforces this concept by confirming that CCN1 can attract and locally immobilize neutrophils, thereby expanding the conventional notion of CCN1 as a cell-matrix adhesion molecule.

## Conclusion

Here, we present evidence establishing a pivotal link between CCN1 and NETs in the progression of DR. By orchestrating the adhesion, migration, and NETs extrusion by neutrophils, CCN1 promotes vascular occlusion, ultimately culminating in capillary degeneration and subsequent retinal leakage (Fig. 8). These findings not only shed light on the etiology of leukostasis in the early stages of DR but also underscore the potential benefits of CCN1 knockdown or the prevention of NETs extrusion in ameliorating retinal leakage in the context of diabetes. However, before advocating for CCN1 as a therapeutic intervention in DR, further investigation is warranted to thoroughly assess any unexpected adverse effects during its clinical application.

### Electronic supplementary material

Below is the link to the electronic supplementary material.

Supplementary Material 1

Supplementary Material 1

## Data Availability

No datasets were generated or analysed during the current study.

## Abbreviations

CCN1: Cellular communication network factor 1
DR: Diabetic retinopathy
DM: Diabetes mellitus
NPDR: Non-proliferative diabetic retinopathy
PDR: Proliferative diabetic retinopathy
NETs: Neutrophil extracellular traps
MPO: Myeloperoxidase
Cit-H3: Citrullinated histone H3
NE: Neutrophil elastase
BRB: Blood-retinal barrier
ELISA: Enzyme-linked immunosorbent assay
IB4: Isolectin B4
STZ: Streptozocin
scRNA-Seq: Single-cell RNA sequencing
OIR: Oxygen-induced retinopathy
rCCN1: recombinant CCN1 protein
LV: Lentivirus
HRVEC: Human retinal vascular endothelial cells
LV-siCCN1: Lentivirus carrying small interfering RNA targeting CCN1
DNase I: Deoxyribonuclease I
AUC: Area under the curve
NOX: NADPH oxidase
ROS: Reactive oxygen species
EB: Evans blue
IL-1b: Interleukin-1 beta
SPF: Specific pathogen free
PFA: Paraformaldehyde
DMSO: Dimethyl sulfoxide
OIR: Oxygen-induced retinopathy
NORM: Normoxia control
GCL: Ganglion cell layer
IPL: Inner Plexiform layer
INL: Inner nuclear layer
OPL: Outer plexiform layer
ONL: Outer nuclear layer
RPE: Retinal pigment epithelium
WBC: White blood cell
RBC: Red blood cell
PLT: Platelet
HGB: Hemoglobin
NEUT: Neutrophils
LYMPH: Lymphocytes
EO: Eosinophils
BASO: Basophils
MONO: Monocytes
NLR: Neutrophil to lymphocyte ratio
NAR: Neutrophil to albumin ratio
TC: Total cholesterol
TG: Triglycerides
LDL: Low-density lipoprotein
HDL: High-density lipoprotein
AST: Aspartate aminotransferase
ALT: Alanine aminotransferase
ALB: Albumin
BUN: Blood urea nitrogen
Cr: Creatinine
CysC: Serum cystatin C
UA: Uric acid
eGFR: Estimated glomerular filtration rate
