# Supplementary material for: Cellular communication network factor 1 promotes retinal leakage in diabetic retinopathy via inducing neutrophil stasis and neutrophil extracellular traps extrusion
Source: Cell Commun Signal. 2024 May 16;22:275. doi: 10.1186/s12964-024-01653-3 (PMC11097549; doi:10.1186/s12964-024-01653-3)
Supplement: Supplementary file 1 — Supplementary Material 1 [file 12964_2024_1653_MOESM1_ESM.docx]

**Supplemental Tables**

| **Variables** | **Non-DM(*N=12*)** | **DM(*N=49*)** | **DR(*N=27*)** | ***P* overall** | ***P^a^*** |
| --- | --- | --- | --- | --- | --- |
| WBC(10^9/L) | 6.20 [4.94;8.01] | 6.43 [5.35;7.23] | 6.60 [5.90;7.69] | 0.701 | 0.504 |
| RBC(10^12/L) | 5.00 [4.58;5.20] | 4.81 [4.37;5.10] | 4.48 [4.04;5.08] | 0.087 | 0.146 |
| PLT(10^9/L) | 216 [194;281] | 198 [174;236] | 245 [170;309] | 0.189 | 0.082 |
| HGB(g/L) | 144 [134;150] | 146 [129;152] | 132 [120;147] | 0.099 | 0.038* |
| NEUT(10^9/L) | 3.21 [2.80;4.26] | 3.44 [2.75;4.32] | 3.69 [3.31;4.72] | 0.238 | 0.118 |
| LYMPH(10^9/L) | 1.84 [1.68;2.29] | 2.15 [1.76;2.51] | 2.02 [1.53;2.30] | 0.531 | 0.359 |
| EO(10^9/L) | 0.16 [0.09;0.24] | 0.14 [0.07;0.27] | 0.14 [0.08;0.23] | 0.837 | 0.996 |
| BASO(10^9/L) | 0.04 [0.02;0.04] | 0.02 [0.01;0.05] | 0.02 [0.01;0.04] | 0.451 | 0.544 |
| MONO(10^9/L) | 0.33 [0.30;0.62] | 0.44 [0.37;0.53] | 0.51 [0.37;0.62] | 0.316 | 0.254 |
| NLR | 1.75 [1.60;1.94] | 1.79 [1.14;2.13] | 1.80 [1.53;2.65] | 0.335 | 0.133 |
| NAR | 0.09 [0.07;0.11] | 0.08 [0.07;0.11] | 0.10 [0.08;0.12] | 0.416 | 0.218 |
| TC(mmol/L) | 4.56 [3.76;4.86] | 4.87 [4.19;5.57] | 4.92 [4.16;5.74] | 0.405 | 0.935 |
| TG(mmol/L) | 1.31 [0.70;1.77] | 1.59 [1.00;2.15] | 1.73 [1.34;2.48] | 0.135 | 0.292 |
| LDL(mmol/L) | 3.28 [2.46;3.67] | 3.04 [2.54;3.81] | 3.12 [2.68;3.70] | 0.945 | 0.724 |
| HDL(mmol/L) | 1.02 [0.86;1.31] | 0.99 [0.90;1.15] | 0.98 [0.82;1.25] | 0.809 | 0.676 |
| AST(U/L) | 20.0 [18.0;26.8] | 21.0 [17.0;28.0] | 19.0 [16.0;28.5] | 0.727 | 0.636 |
| ALT(U/L) | 23.5 [13.0;31.2] | 23.0 [16.0;32.0] | 22.0 [16.0;37.5] | 0.938 | 0.875 |
| ALB(g/L) | 42.8 [40.3;45.7] | 40.1 [37.2;42.7] | 41.9 [39.2;44.1] | 0.227 | 0.195 |
| BUN(mmol/L) | 4.78 [4.10;5.90] | 5.23 [4.61;6.44] | 5.85 [5.20;8.35] | 0.050 | 0.095 |
| Cr(umol/L) | 79.5 [58.7;95.8] | 64.0 [52.0;71.0] | 69.5 [60.1;87.0] | 0.052 | 0.046* |
| CysC(mg/L)T5 | 1.07 [0.78;1.19] | 0.96 [0.84;1.10] | 1.13 [0.96;1.33] | 0.097 | 0.032* |
| UA(umol/L) | 387 [338;443] | 329 [288;407] | 390 [358;456] | 0.027* | 0.008* |
| eGFR | 102 [73.5;114] | 109 [95.8;116] | 97.1 [75.1;104] | 0.009* | 0.002* |

**Table S1. Biochemical measurements of subjects by the presence of DM or DR­**

*P* values < 0.05 indicates the statistical significance and are shown with an asterisk. Results were presented as medians (quartile 1, quartile 3) for not normally distributed variables. Abbreviations: WBC: white blood cell; RBC: red blood cell; PLT: platelet; HGB: hemoglobin; NEUT: neutrophils; LYMPH: lymphocytes; EO: eosinophils; BASO: basophils; MONO: monocytes; NLR: neutrophil to lymphocyte ratio; NAR: neutrophil to albumin ratio; TC: total cholesterol; TG: triglycerides; LDL: low-density lipoprotein; HDL: high-density lipoprotein; AST: aspartate aminotransferase; ALT: alanine aminotransferase; ALB: albumin; BUN: blood urea nitrogen; Cr: creatinine; CysC: serum cystatin C; UA: uric acid; eGFR: estimated glomerular filtration rate. *P^a^*: statistical significance between DM and DR group.

**Table S2. Medications of subjects by the presence of DM or DR­**

| **Medications** | **Non-DM (*N=12*)** | **DM(*N=49*)** | **DR(*N=27*)** | **P overall** | **P^a^** |
| --- | --- | --- | --- | --- | --- |
| Insulin | 0 (0.00%) | 11 (22.4%) | 13 (48.1%) | 0.003* | 0.678 |
| Metformin | 0 (0.00%) | 23 (46.9%) | 15 (55.6%) | 0.004* | 0.922 |
| sulfonylureas | 0 (0.00%) | 7 (14.3%) | 4 (14.8%) | 0.481 | 0.713 |
| Thiazolidinediones | 0 (0.00%) | 0 (0.00%) | 1 (3.70%) | 0.443 | 0.355 |
| AGI | 0 (0.00%) | 9 (18.4%) | 5 (18.5%) | 0.351 | 1 |
| DPP-4 inhibitor or  GLP-1 receptor agonist | 0 (0.00%) | 4 (8.16%) | 6 (22.2%) | 0.090 | 0.09 |
| SGLT-2 inhibitor | 0 (0.00%) | 9 (18.4%) | 6 (22.2%) | 0.212 | 0.506 |

*P* values < 0.05 indicates the statistical significance and are shown with an asterisk. Results were presented as n (%) for categorical variables. Abbreviations: AGI: alpha-glucosidase inhibitors; DPP-4: dipeptidyl peptidase-4; GLP-1: glucagon-like peptide-1; SGLT-2: sodium-glucose cotransporter 2. *P^a^*: statistical significance between DM and DR group.
