# Supplementary material for: Cellular communication network factor 1 promotes retinal leakage in diabetic retinopathy via inducing neutrophil stasis and neutrophil extracellular traps extrusion
Source: Cell Commun Signal. 2024 May 16;22:275. doi: 10.1186/s12964-024-01653-3 (PMC11097549; doi:10.1186/s12964-024-01653-3)
Supplement: Supplementary file 2 — Supplementary Material 1 [file 12964_2024_1653_MOESM2_ESM.docx]

# Supplemental Figures

**
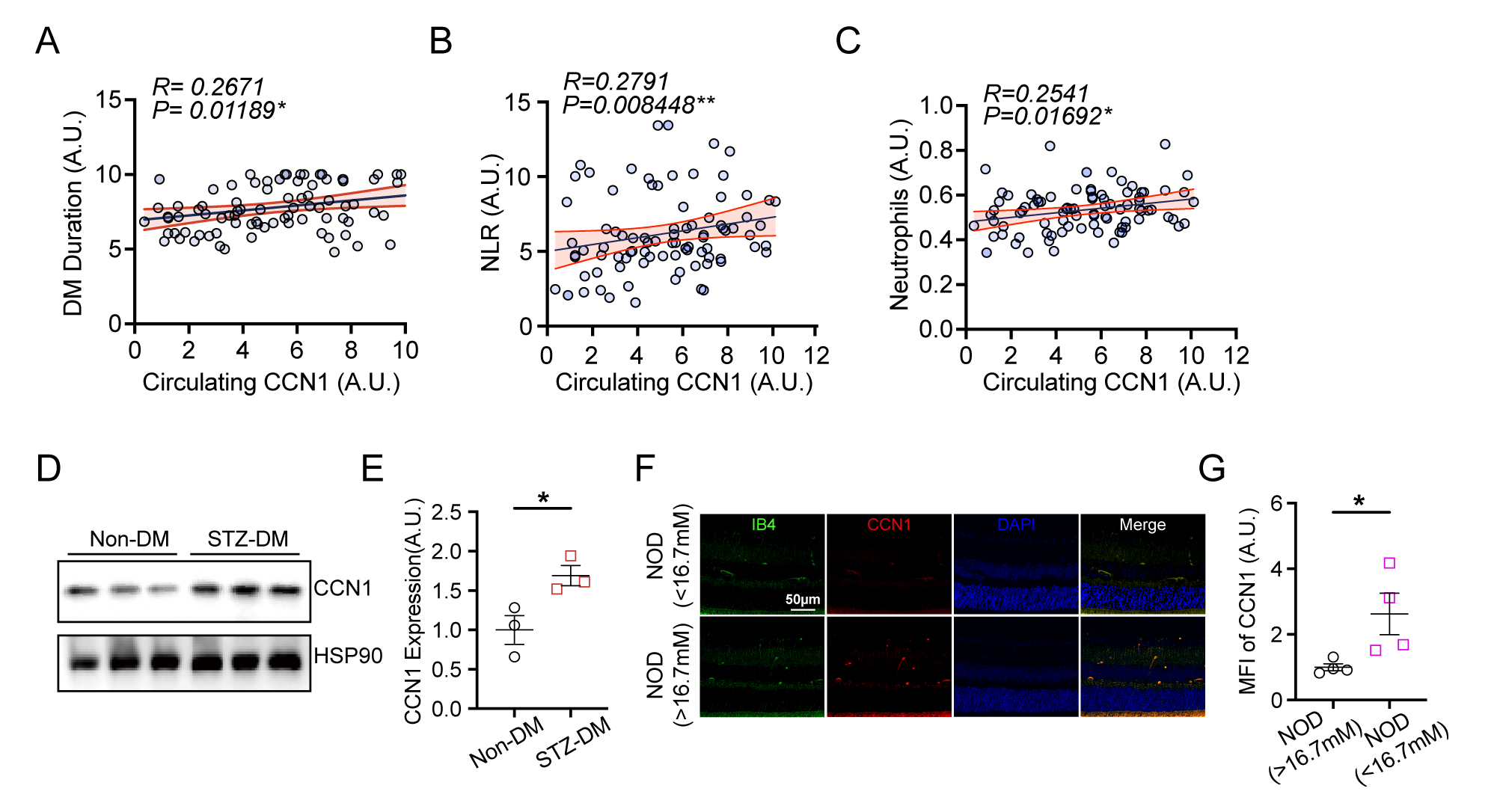
**

**Supplemental Figure 1. CCN1 is increased in diabetic retina.**

(A-C) Linear correlation between circulating CCN1 level and DM duration (A), NLR (B), and neutrophils absolute count (C), correlation was measured using Spearman correlation coefficient (*N* = 88). (D-E) Representative immunoblots (D) and quantification of CCN1 (E) in retina tissue of Non-DM and STZ-DM mice (*n* = 3). (F-G) Representative images (F) and MFI quantification (G) of CCN1 and IB4 on retinas frozen section for NOD mice (*n* = 4). *: *P*<0.05. Data are shown as mean ± SEM. Statistical differences were assessed using unpaired, 2-tailed Student’s t-test.


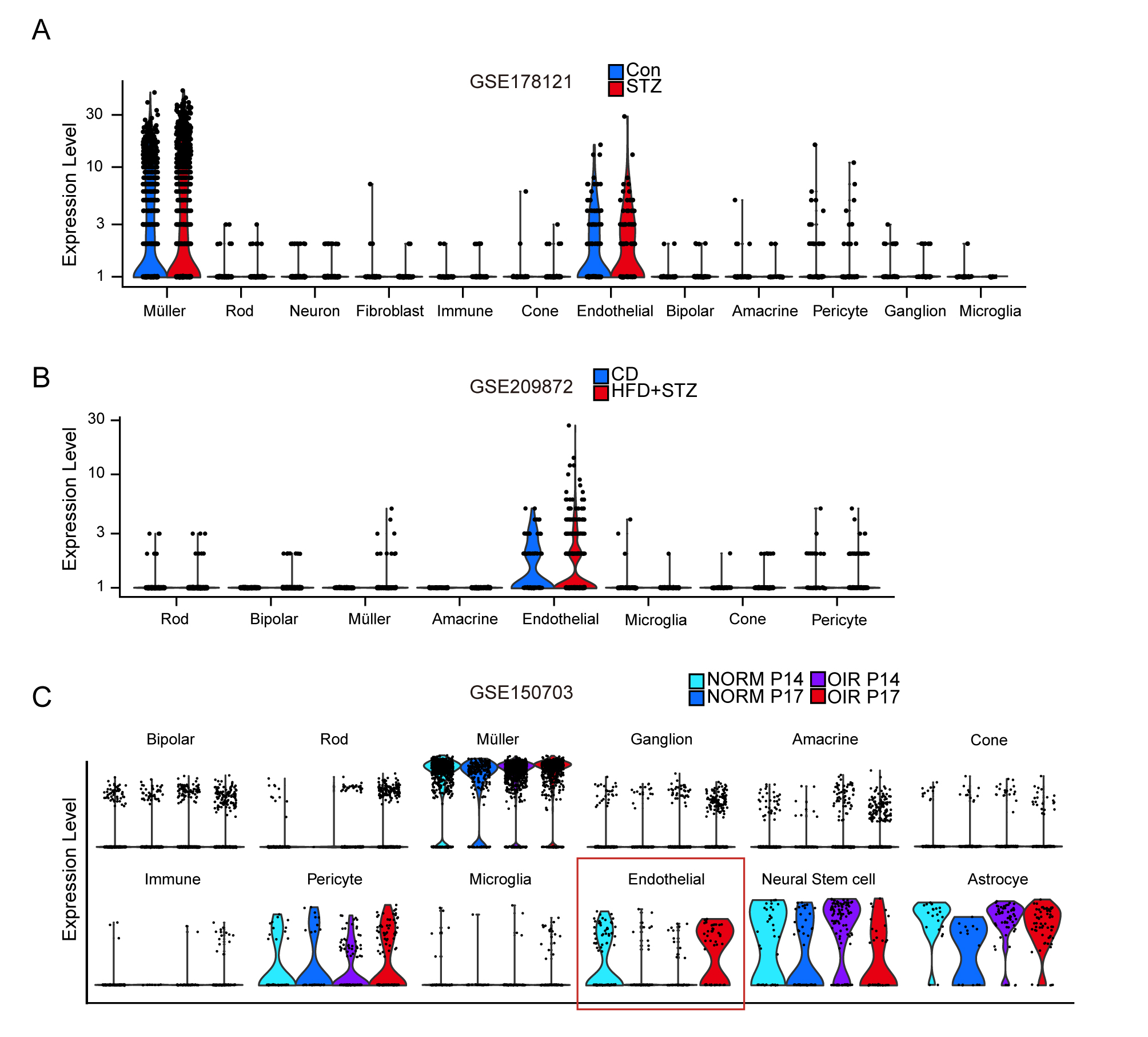


**Supplemental Figure 2. The expression pattern of CCN1 in the retina.**

(A) Violin plots illustrating CCN1 gene expression across various cell types in retina single-cell RNA sequencing data from STZ-treated diabetic mice and control mice (GSE178121). (B) Violin plots displaying CCN1 gene expression in different cell types based on retina single-cell RNA sequencing data from rats fed with high-fat diets and STZ-treated diabetic rats, as compared to control rats (GSE209872). CD: Chow Diets; HFD: High-Fat Diets. (C) Violin plots representing CCN1 gene expression in various cell types as observed in retina single-cell RNA sequencing data from mice with oxygen-induced retinopathy (OIR) and normoxia control (NORM) mice at postnatal day 14 (p14) and p17 (GSE150703).


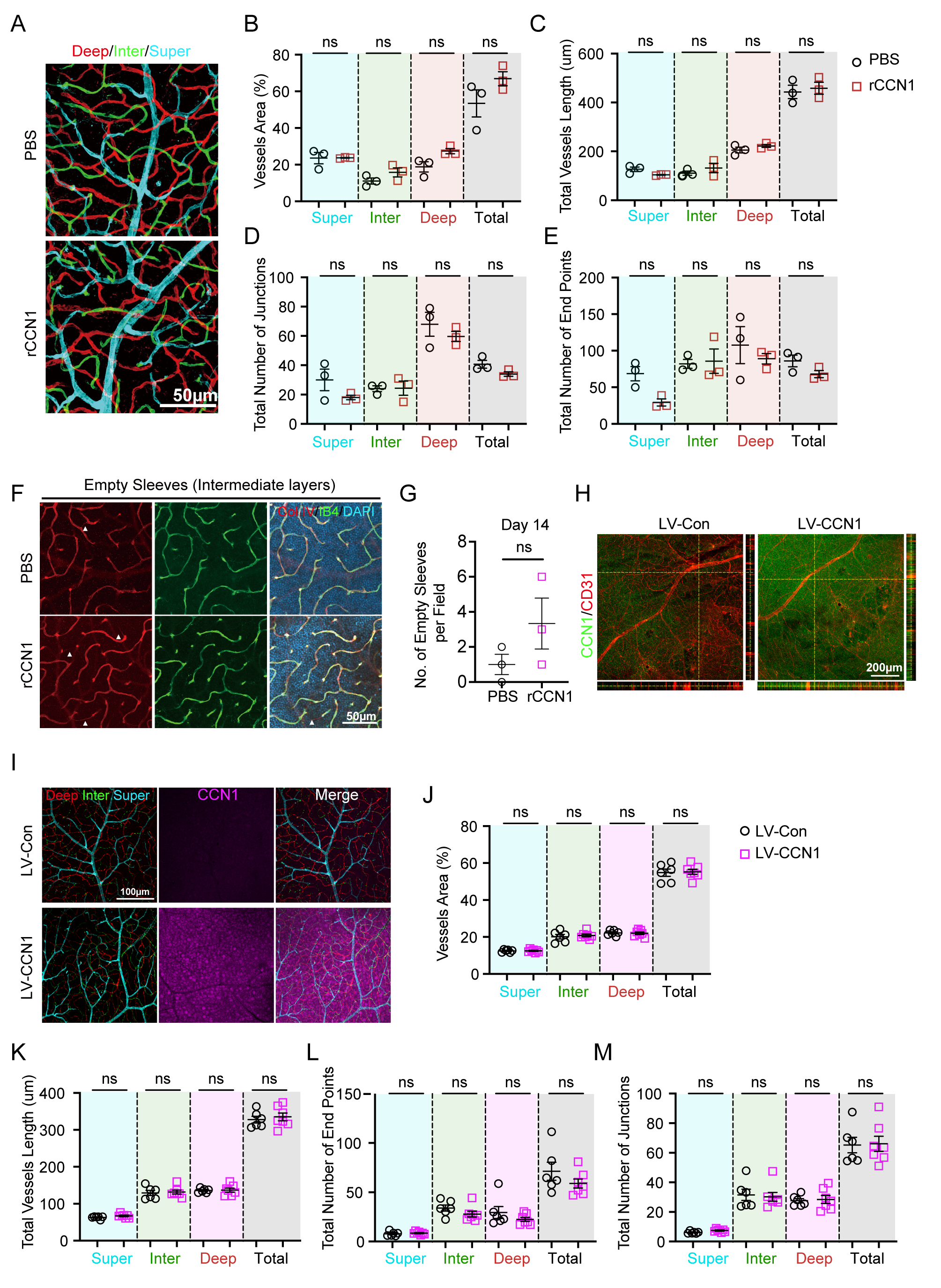


**Supplemental Figure 3. Exogenous CCN1 does not impact retinal vasculature.**

(A-E) Vasculature representative images (A) of deep (red), intermedium (green), and superficial (blue) vessel plexus on day 14 post rCCN1 or PBS injection (*n* = 3), the percentage of vessel area (B), total vessel length (C), the number of junctions (D), and the number of vessel endpoints (E) were analyzed. (F-G) Representative images (F) and the number of empty sleeves (G) in retinal intermedium vessel plexus day 14 post rCCN1 injection (*n* = 3). (H) Representative confocal imaging and 3D reconstruction of retina flat mounts showing a view of the inner retina surface and a z section of the retina stained with CCN1 and CD31 on day 7 post lentivirus injection. (I-M) Vasculature representative images (I) of deep (red), intermedium (green), and superficial (blue) vessel plexus on day 60 after LV-CCN1 or LV-Con intravitreal injection, the percentage of vessel area (J), total vessel length (K), the number of junctions (L), and the number of vessel endpoints (M) were analyzed (*n* = 6 in LV-Con group, *n* = 7 in LV-CCN1 group). ns: no significance. Data are shown as mean ± SEM. Statistical differences were examined by unpaired, 2-tailed Student’s t-test in data.

**
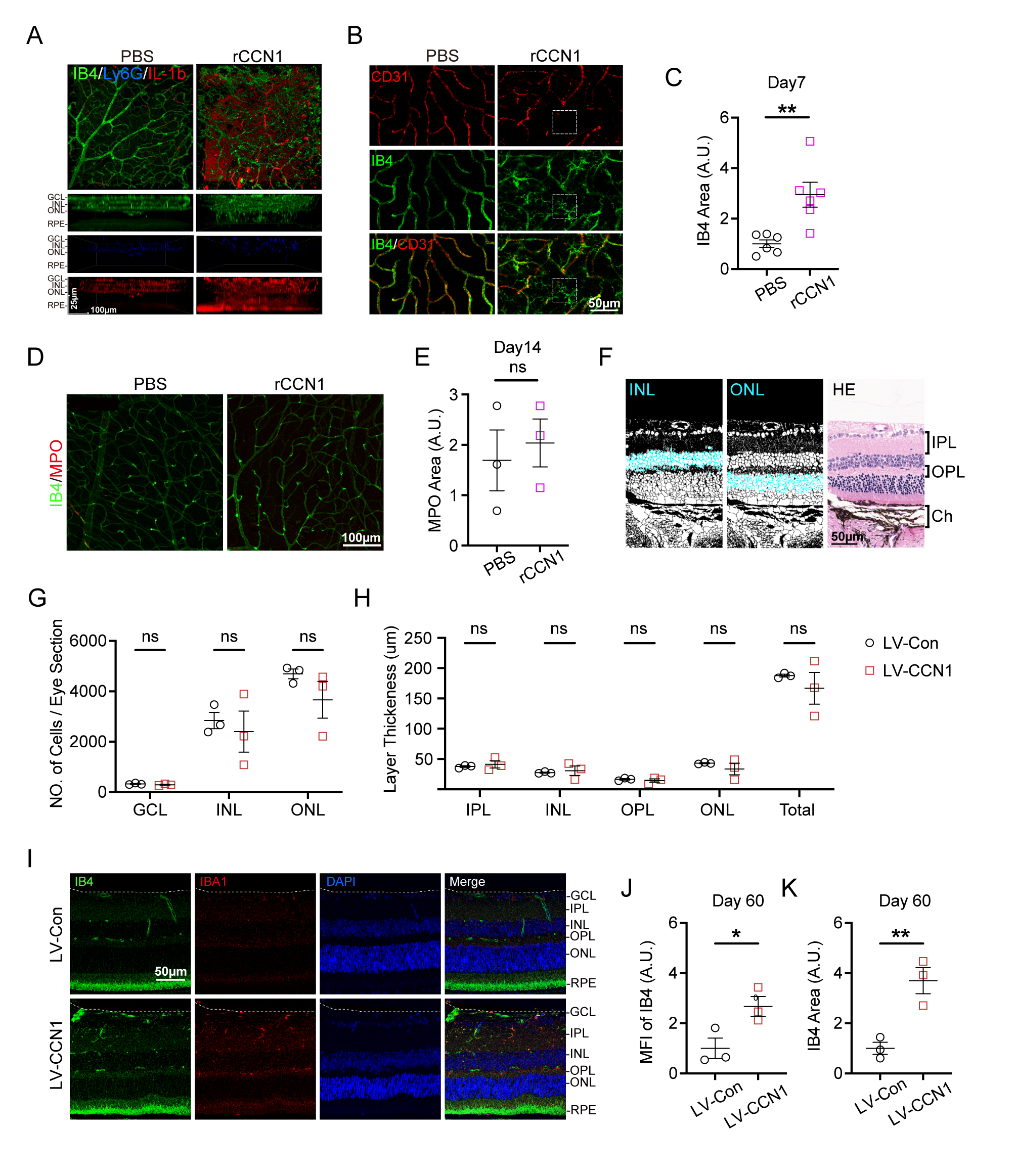
**

**Supplemental Figure 4. CCN1 induces microglia activation and retinal inflammation.**

(A) Representative confocal imaging and 3D reconstruction of retina flat mounts showing a view of the inner retina surface and a z section of the retina with IL-1b, Ly6G, and IB4. (B-C) Representative images of IB4 and CD31 staining (B) in retina flat mount day 7 post rCCN1 injection and quantification of CD31 positive area (C, *n* = 6). (D-E) Representative images of IB4 and MPO staining of the retina (D) and quantification of MPO positive area (E) on day 14 post rCCN1 injection (*n* = 3). (F) Representative H&E staining images of the retina indicate IPL, INL, and ONL. (G-H) The number of neuron cell numbers in GCL, INL, ONL (G), and the thickness of IPL, INL, OPL, ONL, and total retina (H), *n* = 3 per condition. (I-K) Representative images of IB4, IBA1 staining (I), MFI of IB4 (J, *n* = 3), and IB4 positive area (K, *n* = 3) of retina flat mount day 60 post lentivirus injection. ns: no significance, *: *P*<0.05, **: *P*<0.01. Data are shown as mean ± SEM. Statistical differences were examined by unpaired, 2-tailed Student’s t-test.


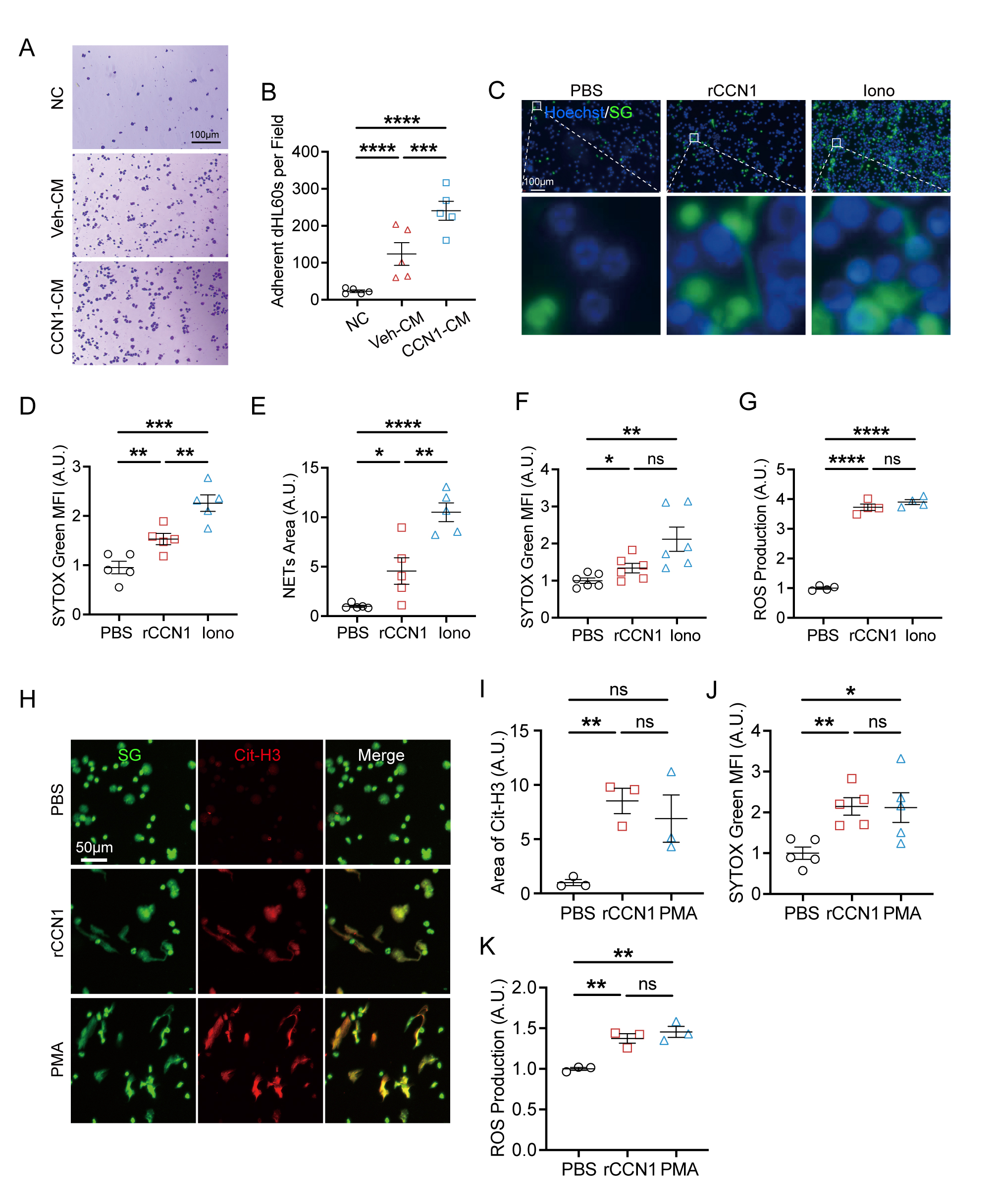


**Supplemental Figure 5. CCN1 mediates the adherence, migration, and NETs extrusion.**

(A-B) Visual assessment of dHL60s adherence to the plate after 4-h co-culture with CM (A) and the number of adherent neutrophils per field (B) was assessed by crystal violet staining (*n* = 5). (C-E) Representative images of Hoechst33342 and SYTOX Green (C), and SYTOX Green fluorescence measurement (D) and quantification of SYTOX Green^+^ area (E) for human primary neutrophils treated with PBS, rCCN1 or Ionomycin (Iono) for 2.5 h (*n* = 5), PBS served as a negative control, and Iono served as a positive control. (F) The SYTOX Green fluorescence measurement for mouse bone marrow neutrophils treated with PBS, rCCN1, or ionomycin for 2.5 h (*n* = 6). (G) ROS production of human primary neutrophils treated with PBS, rCCN1, or Ionomycin (Iono) for 2.5 h (*n* = 4). (H-I) Representative images of mouse bone marrow primary neutrophils stained with Cit-H3 and DNA (SYTOX Green) (H), and quantification of Cit-H3^+^ area (I, *n* = 3). (J) The SYTOX Green fluorescence measurement for mouse bone marrow neutrophils treated with PBS, rCCN1, or phorbol myristate acetate (PMA) for 2.5 h (*n* = 5). (K) ROS production of mouse bone marrow neutrophils treated with PBS, rCCN1, or PMA for 2.5 h (*n* = 3). ns: no significance, *: *P*<0.05, **: *P*<0.01, ***: *P*<0.001, ****: *P*<0.0001. Data are shown as mean ± SEM. Statistical differences were assessed using unpaired, 2-tailed Student’s t-test.


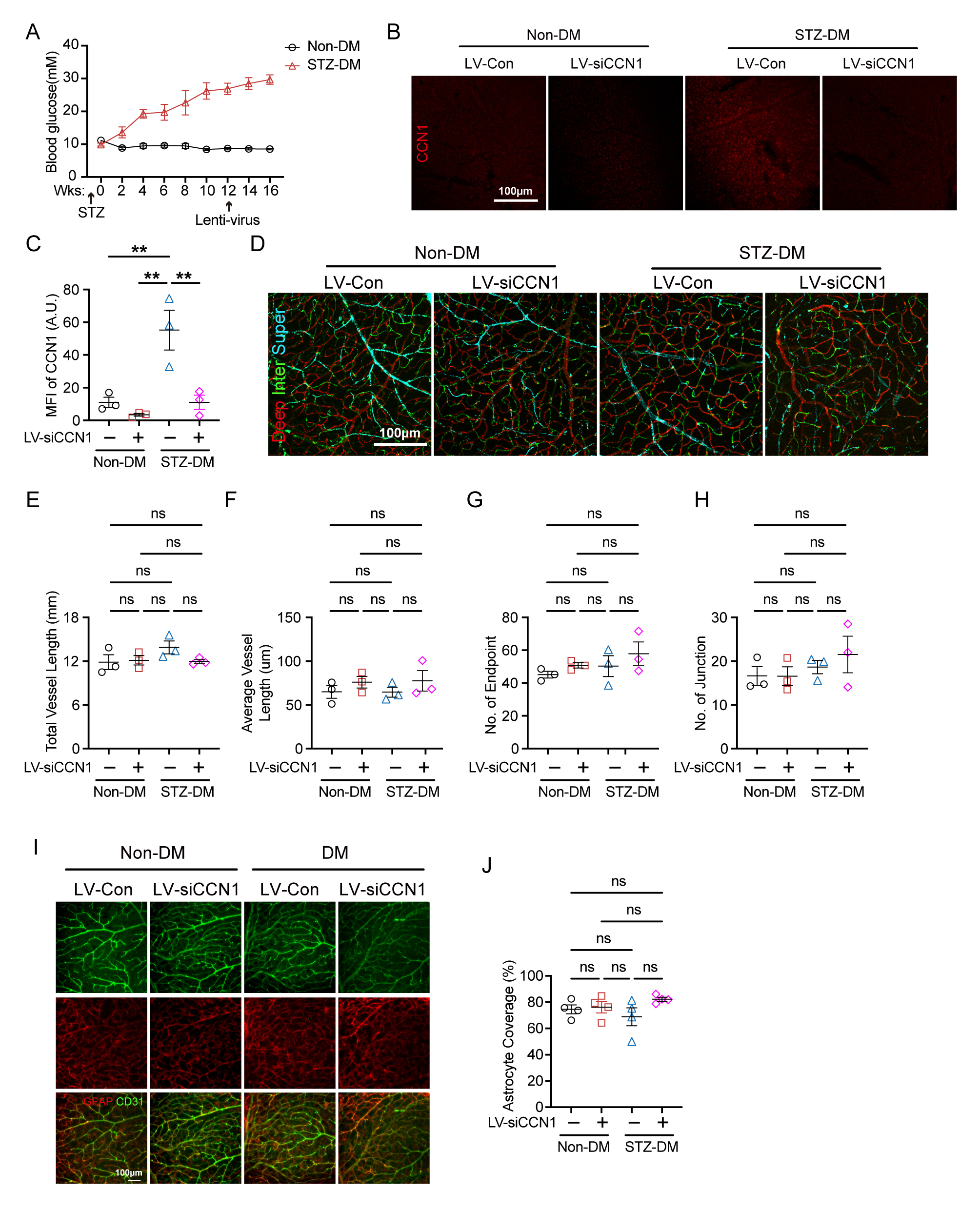


**Supplemental Figure 6.** **CCN1 knockdown in diabetic retina does not cause vessel change in the short term.**

(A) The random blood glucose of STZ-modeling mice. (B-C) Representative retinal flat mount images stained with CCN1 (B) and MFI of CCN1 1 month after lentivirus injection (C, *n* = 3). (D-H) Retinal vasculature representative images (D) of deep (red), intermedium (green), and superficial (blue) layers 1 month after lentivirus injection (*n* = 3), the total vessel length (E), the average vessel length (F), the number of vessel endpoints (G), and the number of junctions (H, *n* = 3). (I-J) Representative images of CD31 and GFAP stained retina flat mount (I) and the percentage of astrocytes’ endfeet coverage around retinal blood vessels (J, *n* = 4). ns: no significance, **: *P*<0.01. Data are shown as mean ± SEM. Statistical differences were assessed using unpaired, 2-tailed Student’s t-test.

**
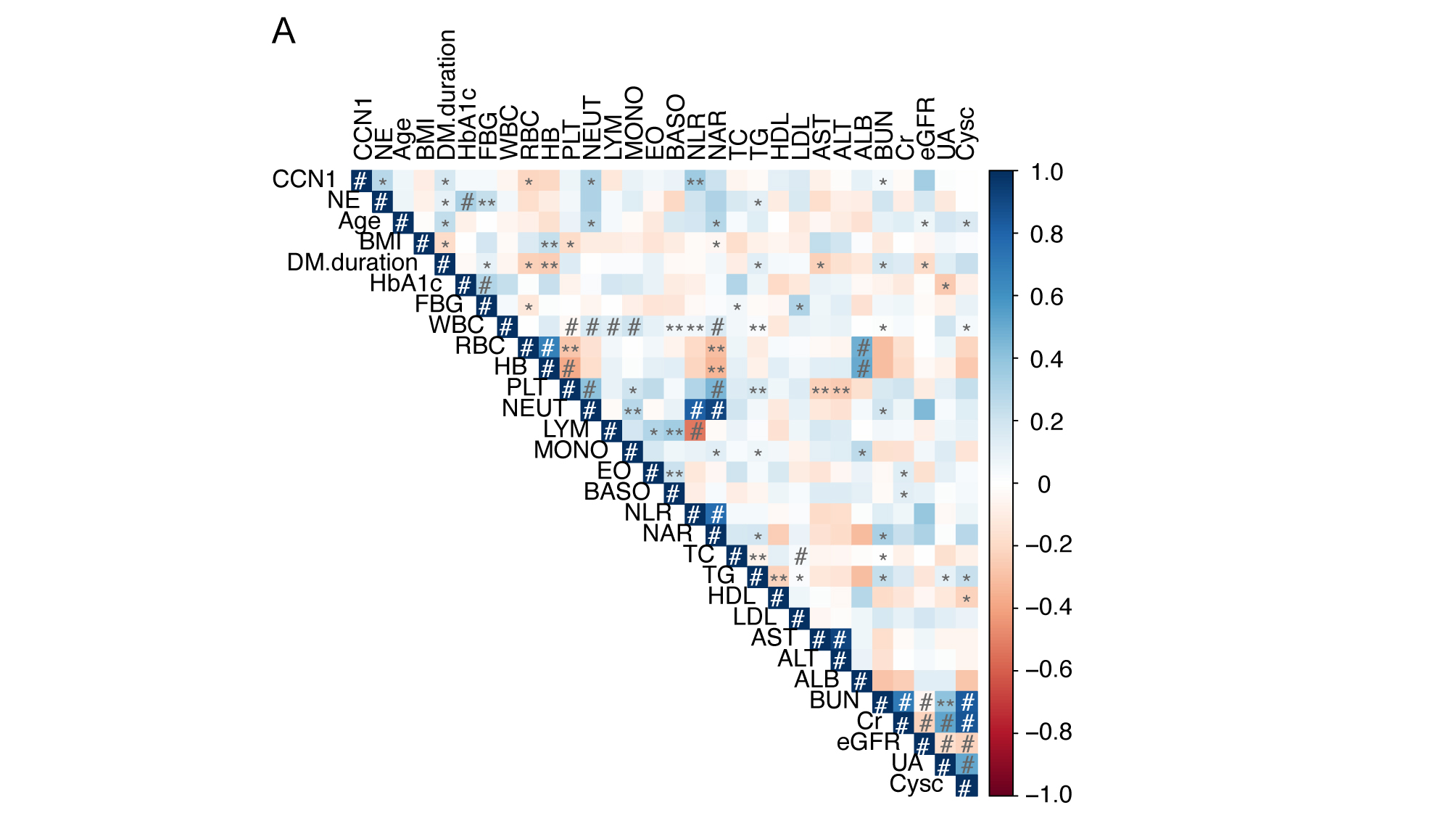
**

**Supplemental Figure 7. Correlation of each index.**

(A) Heatmap of Spearman’s correlation between each test outcome. Red represents the correlation coefficient closer to 1 and blue represents the correlation coefficient closer to -1. *: *P*<0.05, **: *P*<0.01, #: *P*<0.001. The Spearman correlation test was conducted.
